# Nanomedicine and Onco-Immunotherapy: From the Bench to Bedside to Biomarkers

**DOI:** 10.3390/nano10071274

**Published:** 2020-06-29

**Authors:** Vanessa Acebes-Fernández, Alicia Landeira-Viñuela, Pablo Juanes-Velasco, Angela-Patricia Hernández, Andrea Otazo-Perez, Raúl Manzano-Román, Rafael Gongora, Manuel Fuentes

**Affiliations:** 1 Department of Medicine and Cytometry General Service-Nucleus, CIBERONC CB16/12/00400, Cancer Research Centre (IBMCC/CSIC/USAL/IBSAL), 37007 Salamanca, Spain; vanessaacebes@usal.es (V.A.-F.); alavi29@usal.es (A.L.-V.); pablojuanesvelasco@usal.es (P.J.-V.); angytahg@usal.es (A.-P.H.); andreaotazopz@gmail.com (A.O.-P.); rgongora@usal.es (R.G.); 2 Proteomics Unit, Cancer Research Centre (IBMCC/CSIC/USAL/IBSAL), 37007 Salamanca, Spain; rmanzano@usal.es

**Keywords:** nanomaterials, nanomedicine, immunotherapy, oncotherapy, immune-checkpoint inhibitors, immunogenic cell death, nano-vaccines, nano-conjugates, immune response

## Abstract

The broad relationship between the immune system and cancer is opening a new hallmark to explore for nanomedicine. Here, all the common and synergy points between both areas are reviewed and described, and the recent approaches which show the progress from the bench to the beside to biomarkers developed in nanomedicine and onco-immunotherapy.

## 1. Introduction

The broad relationship between immune system and cancer has opened novel therapeutic approaches to treat tumours, such as: monoclonal antibodies, adoptive T-cell transfer, vaccination, immune checkpoint inhibitors, and oncolytic virus therapy. These novel immunotherapies are based mainly on the body’s self-defense system to fight and defeat cancer. Current research is therefore focused on re-activating the immune system to attack cancer cells with potent cytokines, vaccines, antibodies and immune-stimulatory adjuvants. However, these immunotherapies could have several drawbacks, side effects (due to systemic treatment), low efficacy and resistance, among other things. Hence, nanomedicine is a new field with a strong potential application in immuno-oncology in order to overcome the bottlenecks and to improve the current available immunotherapies. Nanotechnology is a new field that has had a great impact on medicine and biomedical research, as it allows for a high-specific targeted delivery to tumour or immune cells, better clinical outcomes and reduces adverse effects, helping the delivery of vaccines and immunomodulating agents. This is made possible by nanoparticles (NPs), which can be highly variable in structure and function. Bearing all this in mind, it seems highly interesting to explore all these fields (nanotechnology, immune-oncology, immunotherapy, nanomedicines, etc.) in order to find and discover synergies and new opportunities; thus, here, the major features and achievements in these areas are briefly reviewed.

## 2. Nanomedicine

Nowadays, nanomedicine is an emerging and highly relevant area due to the fact that great advances have been made in the treatment of various diseases, such as cancer, neurodegenerative and cardiovascular diseases, and hormonal problems. To understand the development and possible applications of nanomedicine, it is necessary to define the concept of nanotechnology.

### 2.1. Nanotechnology: Brief Description

Nanotechnology can be defined as the “development of science and technology at atomic and molecular levels, at the scale of approximately 1–100 nm, to obtain a fundamental understanding of phenomena and materials at that nanoscale and to create and use structures, devices and systems that have new properties and functions because of their size” [1].

Nanotechnology has been emerging in science and technology for the last 20 years. When working at this scale, matter undergoes radical changes in its physical and chemical properties, such as in electrical conductivity, colour, and resistance or elasticity, giving it interesting properties that can be used in many applications in different fields, including electronics, medicine, engineering, environment and energy [1,2]. There are many studies describing a wide number of current nanotechnology applications in multiple fields, such as oil recovery, the formation of conductive films that can be used in electronic devices or even improving anaesthesia in medicine, as just a few examples that illustrate the broad fields of applications [3,4,5,6].

### 2.2. Nanomedicine: Concept

The application of nanotechnology in the health sciences has given rise to nanomedicine, a new discipline that aims to develop tools for diagnosing, preventing and treating diseases at an early stage of their development [1]. 

Nanomedicine is an interdisciplinary field in which nanoscience, nanoengineering and nanotechnology interact with the life sciences. It is expected that nanomedicine will lead to the development of better devices, drugs and other applications for early diagnosis or treatment of a wide range of diseases with high specificity, efficacy and personalization with the aim of improving the quality of life of patients. Because of its broad scope, it is expected that nanomedicine can be involved in all aspects of medicine, i.e., enter into conventional clinical practice. Nanomedicine differs from other types of conventional medicines in that it involves the development and application of materials and technologies with nanometric length scales [7]. 

Nanomedicine covers three main areas: nanodiagnosis, controlled drug delivery (nanotherapy), and regenerative medicine. All these areas are briefly described below [1].

Among other nanotechnology strategies, NPs are the key component that allows the development of nanomedicine, and currently there is a great variety of them. The properties of these NPs are affected by their size, shape, and surface bio-functionalization which is relevant for the characterisation of the NPs for each particular medical application. This comprehensive characterisation and precision synthesis allow for these NPs to perform specific functions and these functions can be correlated with specific characteristics of the NPs. In addition to characterization, the development of new methods of separation and purification of NPs is also needed to produce optimal samples for nanomedical applications and to study the behaviour of NPs within biological proximal fluids (serum/plasma, etc.), cells, tissues and the human/animal body. Despite these drawbacks to overcome, NPs are expected to improve the detection and early diagnosis of diseases, and also to help to provide personalised medicines [7].

**Figure 1:**
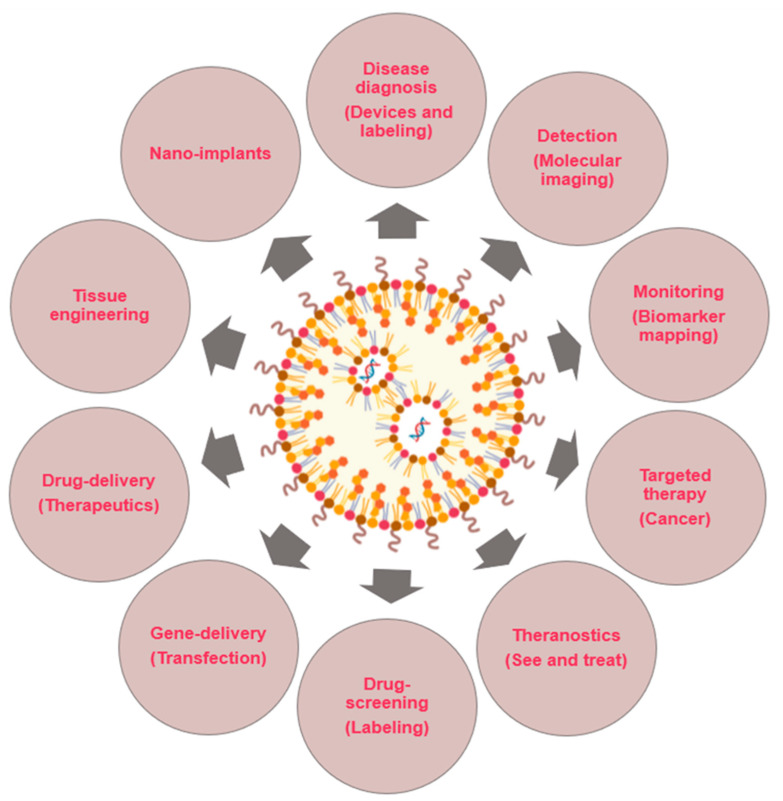
Diagram displaying multiple applications of nanotechnology in Medicine.

NPs have a wide range of applications in nanomedicine (Figure 1). NPs can be designed to provide contrast at the targeted zone and report information about the local environment after administration into the body, which also offers the possibility to label tissues with selected markers and enables the local read-out of concentration of targeted molecules, which helps to analyse diseases directly inside the human body. Another application of NPs consists of the in vitro analysis of human proximal body fluids (such as ones of the major sources for biomarkers), participating in massive diagnostic strategies with the aim of detecting molecular alterations. Through NPs, multiple biomarkers can be analysed simultaneously, improving diagnostic accuracy and reproducibility [7].

NPs are also used for the treatment of diseases, either as drug delivery vehicles, as bioactive materials or as components in implants [8,9]. In addition, nanomedicine is being implemented in the development of new matrices, support or surfaces for the design of implantable and electronic sensors or systems to aid in tissue regeneration; i.e., NPs are beginning to be used in regenerative medicine [7].

Here, several highlights of the major interested areas (nanodiagnostic, targeted drug release, regenerative therapy) about this topic covered by nanomedicine are briefly described.

#### 2.2.1. Nanodiagnostics

In general, nanodiagnosis is considered as the design and development of analytical and imaging systems that allow for the detection of disease or abnormal cell function in early stages, both in vivo and in vitro [1].

**Figure 2:**
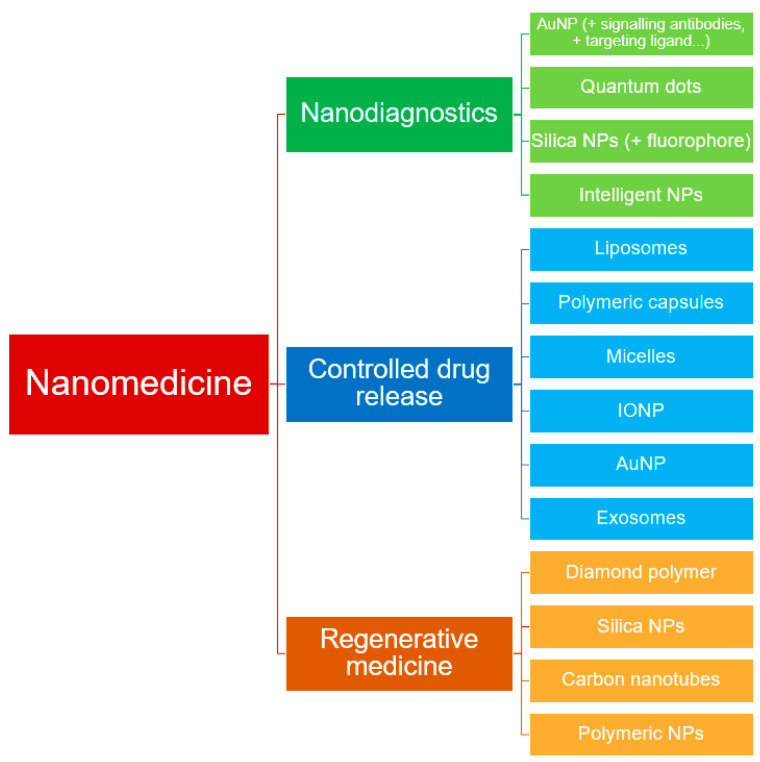
Current nanoparticle (NP) involvement in the multiple applications of nanomedicine.

Nanomaterials can be used for in vivo diagnosis, being used as contrast agents to visualize tissue structures inside the human body and to delimit healthy vs. pathological tissues. To this end, NPs are designed with different contrast properties for different modalities, such as computed tomography (CT), magnetic resonance imaging (MRI), positron emission tomography (PET), single photon emission computed tomography (SPECT) or fluorescence imaging. NPs will be designed to target specific tissues and generate the contrast. Then, to illustrate the applications, some of these examples are described below (Figure 2) [7].

In the case of CT, X-ray imaging takes advantage of tissue-specific attenuation to generate contrast on X-rays screenings, i.e., bone generates more contrast than soft tissue due to a higher relative electron density in the bone. To increase the contrast of these soft tissues, elements such as iodine or barium, which have a high electron density, were used, but to increase the low sensitivity, NPs were developed as contrast agents [7]. Among these NPs, AuNPs, which have a high electron density, stand out [10]. AuNPs have directional ligands like folic acid to bind to different tissue structures through their corresponding receptor composed of other types of materials that have a high atomic number are also suitable for CT. NP-based CT imaging technologies may change the way clinical diagnosis based on CT is performed [11]. In the case of iodine or barium, the doses required are very high, the contrast agents are usually non-specific and do not bind to cellular biomarkers or accumulate in tissues of interest, so the aim is to design NPs with high atomic number materials conjugated with targeting molecules that allow for different cell types to be specifically marked in vivo [7].

In the MRI example, contrast agents based on biocompatible NPs have advantages over the conventional contrast, such as the ability to adapt their size, shape, composition, circulation time, target cells, and optical and physical properties to optimize the images [7].

There are “smart” NPs that are activated by certain stimuli, such as pH, temperature, redox reactions, ions, proteases or light. These NPs respond to a change in the tumour microenvironment (TME) and allow for the selection of the diagnostic and therapeutic mechanism, which is highly relevant in oncology, because the TME regulates the progression of the tumour and its metastasis. In the case of MRI, probes of these “smart” NPs have been designed that are sensitive to pH, since it is a very important physiological parameter and its deregulation might be a biomarker of cancer. Additionally, hypoxia in the TME results in the production of lactic acid and therefore in acidic conditions, which also constitute a Damage-Associated Molecular Pattern (DAMP). Other probes of these types of NPs used in MRI are the temperature sensitive ones, since in tumours, differences in temperature between tissues are very common [7].

NPs could also be used for in vitro diagnosis, i.e., the detection of molecules, cells and tissues outside the human body. In this case, the function of NPs is to identify unique biological molecules in biological fluids that are associated with the health of patients and are useful for diagnosis. In this case, NPs are coated with ligands and biomolecules to allow for bio-recognition of biological molecules in such fluids [7]. Following the example of AuNPs, in this case they are modified with ligands that bind to a specific complementary protein, causing the agglutination of these NPs, which can be observed colorimetrically [12]. This knowledge has also been used in the detection of colorimetric DNA. The AuNPs diagnostic technique is used in the clinic to analyse patient samples [13]. Hence, AuNPs also serve as biosensors, conjugated with antibodies against signalling proteins, such as anti-CA15-3-HRP, to test CA15-3, which is an important tumour biomarker for breast cancer follow-up. The use of magnetic NPs as proximity sensors in MRI is known as diagnostic magnetic resonance imaging (DMR) [2,14,15].

Another example is the use of QD as fluorescence markers in proteins or nucleic acid assays, such as the detection of antigen surface epitopes [16]. Organic and inorganic polymer NPs have been used in intracellular detection applications. An example is silica NPs carrying fluorophores for intracellular detection of oxygen, pH or metal ion levels [17].

#### 2.2.2. Controlled Drug Release

Bearing in mind the complexity, the conventional drug delivery system cannot deliver the chemotherapeutic agents in the most effective concentration to cause tumour cell death, and debilitating side effects occur. This has led to the development of NPs as a drug delivery system (Figure 2), with the aim of achieving tumour specificity and improving the therapeutic index and pharmacokinetic profile of chemotherapeutic agents [18]. Thus, nanotherapy may allow for target active nanosystems containing recognition elements to act or transport and release drugs specifically on affected areas or cells, with the goal of achieving more effective treatment with fewer side effects [1].

Although NPs have been designed to treat various diseases, their most important application has been in cancer. Many of the NPs formulations for cancer treatment have already been approved by regulatory agencies and used in the clinic, but although they produce fewer adverse effects than naked drugs, their therapeutic effectiveness sometimes does not improve substantially. Therefore, the objective is to develop systems with greater therapeutic efficacy [7].

For nanomedicine to have a high therapeutic efficacy in the administration of drugs against cancer, it must comply in the most efficient way with the five steps of the CAPIR cascade: blood Circulation, Accumulation and Penetration in the tumour, cell Internalization and intracellular Release of the drug (CAPIR) [19]. The current approach to nanomedicine development is to adapt the basic physicochemical properties of NPs (size, surface properties and stability, among others) to achieve the CAPIR cascade. As a consequence of the enhanced permeability-retention effect (EPR), it has been proven that passive diffusion allows for tumour localization of nano-chemotherapeutics, but within the TME the localization of nano-chemotherapeutics can be obstructed by different parameters, such as high interstitial fluid pressure, altered extracellular matrix structure, increased cell division or altered lymphatic drainage. Therefore, there is a need to understand the barriers of TME and modulate it to improve the delivery of these drugs [18].

Different types of available NPs are suitable as drug delivery vehicles, which can be passively or actively targeted at tumour tissues to improve the selectivity of these drugs and reduce their side effects. One of the FDA-approved delivery vehicles is liposomes, which are already used in several cancer therapies (i.e., Doxil) [20]. Polymer nanocapsules, which are made of completely hydrophilic polymers, are used to encapsulate hydrophilic drugs. Polymeric micelles are also used for drug delivery, which involves the self-assembly of amphiphilic molecules. The encapsulation of the anti-tumoral drug in these micelles reduces toxicity and improves circulation [7]. An example is the loading of cisplatin into micelles formed by polyethylene glycol (PEG), which increases the time of drug circulation by reducing acute renal accumulation of polymeric micelles [21].

Platinum-derived anti-cancer drugs are of great use, applied in the treatment of cancer, and now a few of them are back in the spotlight because of the recent developments of onco-immunotherapy. In the study conducted by Díez P. et al., a bile-cysplatin acid derivative conjugated to IONPs (iron oxide NPs) was obtained that improves selective cytotoxic activity and promotes the usefulness of IONPs as drug carriers in tumoral cell lines, where platinum derivatives have shown low efficacy. The use of these IONPs may be of great interest in cancer therapies, as they can be designed to bind tumour cells and release the drug in a specific way [22].

Gold-NP, polymer NP or liposomes are also used as carriers of tumour-peptide vaccines that play an important role in tumour immunotherapy [2,23,24]. Chemotherapy based on platinum (II), ruthenium and gold (III) compounds also kills tumour cells [25,26]. One of the most studied gold (III) compounds is the anti-rheumatic drug Auranofin as a cancer treatment [2,27].

Another type of structure involved in nanomedicine are the exosomes, which are naturally occurring nanosized vesicles secreted endogenously by the cells themselves [28]. They are involved in intercellular and tissue-level communication through the transfer of biological material between cells. Exosomes have great potential for use as nano-carriers for various therapies in both inflammatory diseases and cancer, as well as for diagnosis [7].

In general, for controlled drug release, NPs must be designed to escape immune clearance, but they must also be able to adhere to the target tissues and be absorbed or interact with the desired cells in vivo. They can accumulate in the tissues actively or passively, either through transport by intra-organic pressure or through adhesion to specific biological structures in the target tissue by recognition of surface-bound ligands by molecules [29]. In addition to adapting the surface properties of NPs, the optimization of NPs size is also necessary for their accumulation and penetration into tumours and to ensure treatment efficacy [7]. In addition to passive targeting, the active targeting of NPs is also being developed. One example is the design of integrin-targeted nanomedicines using RGD-modified liposomes, which have been shown to result in elevated intracellular levels of doxorubicin [30]. In this sense, novel ligands are being developed against tumour targets, using different targeting biomolecular motifs. There is still discussion about the benefits of active versus passive targeting [31]. Many different controlled release systems are also being developed, which selectively control the rate of drug release by acting on the diseased cells [8].

Another alternative delivery strategy is the combination of multiple antitumour drugs in a single carrier [32]. Co-administration of chemotherapeutic drugs and nucleic acids has led to promising results in overcoming resistance to multiple drugs. Combining therapies against more than one tumour target improves the therapeutic outcome [33]. One of the advantages of nanomedicines is that they can be administered locally, unlike most chemotherapeutics, which are administered systemically.

#### 2.2.3. Regenerative Medicine

Regenerative medicine aims to repair or replace damaged tissues and organs using nanotechnology tools [1]. Nanomaterials designed to deliver drugs or perform some action on diseased tissue are programmed to degrade later, but nanomaterials that are not removed and remain performing their function continuously are also being synthesized. These nanomaterials will allow for surface modelling and provide new functions in tissue engineering, such as new properties of implants (Figure 2). One example is carbon nanostructures, which are biocompatible and support the growth and proliferation of different cell types [7].

Diamond polymer composites are used in implant nano-engineering, which have the potential to restore damaged tissue [7]. They have very good mechanical properties, which together with the administration of drugs and biological molecules and their biocompatibility, allow for the re-enforcement of implantable polymers, creating the support of multifunctional tissues [9]. Furthermore, they are non-toxic and their production is scalable.

For the application of these types of implants, the interface between the implanted devices and the surrounding cells and tissues is also important. This is where the geometry of the selected device comes into play [7]. Another application is found in neuronal systems, where carbon nanotubes (CNT) are used, which influence the electrical activity of the neurons by improving neural signalling, inducing the formation of a greater number of synaptic contacts and promoting the growth of nerve fibers [34,35,36].

Biological implants, such as cell-based therapies, are also of great importance in regenerative medicine. One example is the administration of stem cells to regenerate defective tissue [37]. Here, nanotechnology helps to create culture substrates that enable the adhesive properties of the cells to be activated and de-activated. Nanotechnology is also being used in the engineering of artificial organs for regenerative medicine [7].

Nanoconstructions can also be used to control or lead directly cell behaviour, such as nanoscale silicate materials that induce targeted differentiation of mesenchymal stem cells (MSCs) in osteogenic targets [38]. Polymer NPs can be used to release growth factors and cytokines in a controlled manner, such as the release of angiogenic factors (CEGF and PDGF) that induce blood vessel formation [7].

With a better understanding of how nanoscale devices interact with cells, together with the ability to design more controllable nanomaterials, a new era of nanomedicine can be reached for applications in regenerative medicine.

### 2.3. Nanomaterials in Medicine

**Figure 3:**
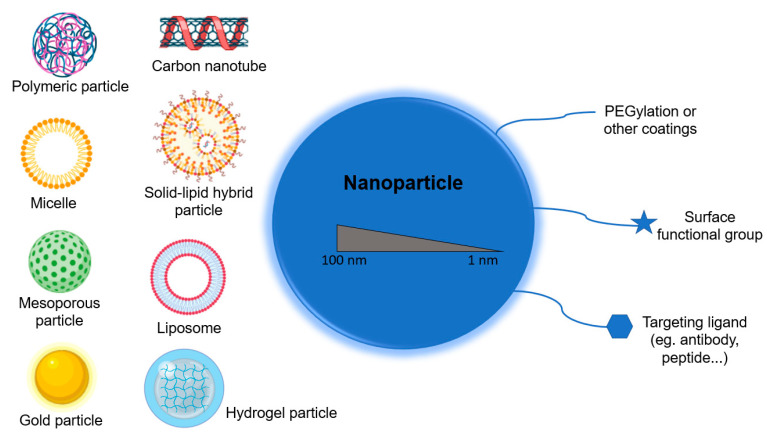
Nanomaterials currently used in the design of NPs, and the available surface modifications.

At the nanoscale level, properties exist in all materials, both natural and synthetic, but only synthetic materials are generally considered to be part of “nanoscience and engineering” [7]. A wide variety of NPs are currently available, and many of the nanomaterials used can mimic the functions of globular biological macromolecules. These materials include lipid micelles, polymer nanostructures, protein constructions, ribonucleic acid NP, carbon dots, nanodiamonds, carbon nanotubes, graphene, and some inorganic materials such as mesoporous silica NP, superparamagnetic iron oxide NPs, and quantum dots. (Figure 3) [39,40,41,42,43,44]. These types of materials have unique optical, electronic and magnetic properties depending on size and shape [45].

In recent years, the understanding of MSD-mediated immunotherapy in cancer treatment has improved and a variety of nanomaterials have been developed to regulate MSD. The following is a description of multiple types of NPs composed of a variety of nanomaterials that are used to enhance some of the immunotherapies that are discussed in more detail in this review.

In the case of nanovaccines, for example, the size of these NPs is associated with the mechanism of cellular absorption and the subsequent endocytic pathway, which in turn determines the effect and outcome of the NPs on the cells. The smaller PNPs (25–40 nm) drain into the nodes through the tissue barrier faster than the larger NPs (100 nm), which have to be transported by dendritic cells (DCs). The shape of the NPs is also important in cellular uptake and bio-distribution [46]. Non-spherical NPs have been shown to prevent non-specific cellular phagocytosis by prolonging their systemic circulation, but spherical NPs are more easily transported by DCs [47]. Another important parameter is the charge of NPs, since it influences their internalization and further induction of immune response. Cationic NPs are absorbed more rapidly by macrophages or DCs and have a higher lysosomal escape potential, but they adsorb more serum proteins, reacting with negatively charged components, reducing the permeability of tumour tissues. The NPs that have the better circulation and best penetration into tumours are neutrally net charged NPs [48].

One of the most promising NPs are biodegradable NPs, which generally use poly (lactic-co-glycolic acid) (PLGA), which also has the advantage of a protective effect on antigens [49]. The size of these NPs is the same as that of pathogens, so they are better absorbed by antigen-presenting cells (APCs).

Inorganic and metallic NPs are also used as nano-vaccines. In this case, functional ligands are conjugated with mesoporous silica, calcium phosphate and gold NPs. Peptide micelles, dendrimers, oncolytic viruses and artificial exosomes are also being developed as DC-based nanovaccines [46].

Another type of NPs that allow for the improved recognition of TSAs by the immune system are polymeric NPs that contain large amounts of adjuvant and are membrane-coated by tumour cells with various types of TSAs [50]. Then, depending of properties of polymeric NPs and the type of immunotherapies, several applications have been developed which here are briefly described: i. In the case of aAPCs, dextran-conjugated superparamagnetic iron oxide NPs with major histocompatibility complex (MHC)-Ig dimer and anti-CD28 antibody are used. Magnetic field-induced aAPCs stimulate the activation and proliferation of antigen-specific T-lymphocytes [46]. ii. For cellular immunotherapy, polyNPs (β-amino ester) with a CAR-coding plasmid DNA load are used to enhance chimeric antigen receptor-modified T cells (CAR-T) cells [51]. iii. As for checkpoint inhibitors, zinc pyrophosphate (ZnP) NPs loaded with photosensitizing pyrolipid (ZnP @ pyro) for photodynamic therapy (PDT) have been shown to improve tumour sensitivity to PD-L1 (programmed death-ligand 1) blocking immunotherapy and induce immunogenic cell death [52]. iv. For cytokines, NPs with a self-assembly derived from PEGylated polylactic acid and cationic phospholipid have been designed for targeted administration of IL-12 plasmid DNA [53]. v. Another example is directed AuNPs loaded with endostatin, which blocks neovascularization and normalizes tumour vasculature [54].

Polymeric nano-carriers are used to deliver adjuvant, which accumulates at the site of the tumour through permeability and retention. An example is the use of polyethylene glycol (PEG)-PLGA NPs to encapsulate R837 and a near-infrared dye via an oil-in-water emulsion [55]. PLGA NPs are also used to improve the supply of monoclonal antibodies (mAb) and enhance the activation of T cells [56]. An example is the chemical conjugation of mAb against OX40 (tumour necrosis factor receptor) with PLGA NPs [57].

Another polymer under study is acetylated dextran, which enhances the properties of traditional polymers by allowing for the loading of hydrophilic drugs in a very efficient way, and it is biodegradable and pH-responsive, dissolving under acidic conditions but remaining stable under physiological conditions [56].

Liposomes are also nano-carriers, which allow for a more specific delivery of cytokines and mAb to the site of the tumour. The payloads can be conjugated on the liposomal membrane or charged in the center of the particle. An example is IL-2 and anti-CD137 sticky liposomes [58].

Water-in-oil emulsions are also used, which are large in size and provide a reservoir for the local release of therapeutic agents [56]. An example is the use of these water-in-oil emulsions to deliver anti-CTLA-4 antagonistic antibodies and anti-CD40 agonist antibodies [59].

Another type of material used is hydrogels, which are particularly suitable for delivering biomolecules [56]. They can be generated by the self-assembly of amphiphilic polysaccharides, and cholesterol-bearing pullulan (CHP)-based platforms are also being studied in immunotherapy [60]. Hydroxypropyl cellulose (HPC) nanogels have been shown to drain nearby lymph nodes after skin administration and release their antigen payload into the APCs, enhancing antitumour immunity [61]. Another example is the bioreducible cationic alginate-polyethylenimine nanogel, used to encapsulate ovalbumin as a vaccine that is absorbed by dendritic cells, facilitating antigenic presentation and activating immune responses [62]. Nanogels can also be used in the administration of cytokines, such as murine IL-12 that is incorporated into a CHP nanogel, allowing its sustained release into the bloodstream [63].

AuNPs show great promise due to their safety and adjustable nature, and increase the potency and decrease the toxicity of immunotherapeutics through improved patency and retention [56]. AuNPs conjugated to a tumour peptide that binds to CD13 in the tumour endothelium have been shown to transport and release TNF-α more effectively in vivo [64]. AuNPs can also be used as contrast agents in CT. As an example, the administration of anti-PD-L1-conjugated AuNPs in mice generated a CT signal that correlated with tumour growth, so these NPs can be used to predict responses to immunotherapy treatments [65].

Because of their porous structures, mesoporous silica NPs (MSNs) have a high intrinsic payload encapsulation capacity [56]. An example is the use of liposome-coated MSNs loaded with doxorubicin and oxoplatin (apoptosis inducers) and indoximod (an adjuvant that interferes with immunosuppressive pathways in MSDs), increasing their half-life in circulation and tumour targeting [66]. MSNs have also been designed with large pores that induce a potent immune response when it is combined with photothermal agents and model antigens [56,67].

Other nanoplatforms that are starting to be used are biomimetic nano-carriers, which further improve delivery efficiency and subsequent immune responses. Natural debris can be used to design these NPs, modifying their surface and improving their absorption by the target cells [56]. An example is mannose modification, which has an affinity for receptors present in APC [68]. Galactose modification is another example of biomimetic targeting [69]. These natural carriers also include virus-like particles (VLPs), e.g., cowpea mosaic virus (CPMV)-based VLPs, which combined with an antigenic peptide of human epidermal growth factor receptor 2 (HER2) protein can be used as a vaccine in the treatment of cancer of HER2+ tumours [70].

Heat shock proteins (HSP) also interact with APC receptors and improve antigenic presentation. An example is the use of HSP96-bound antigenic peptides, which are used as a vaccine in colorectal liver metastases [71].

Lipoprotein-based nanoporters are also used, such as the synthetic high-density lipoprotein-mimicking nanodisc that has been used in the targeted vaccination of neo-antigens [72].

Briefly, delivery platforms and their biomimetic modifications provide different advantages in cancer immunotherapy. In addition, many of these nanoplatforms are located at the interface of the natural and the synthetic nanomaterials. Despite the advantages, there are several challenges for these nano-carriers, which include the cost-effective supply of biological nanomaterials, their large-scale production at the pharmaceutical level and the optimisation of long-term storage conditions [56].

The great development of these nanomaterials and the importance they have acquired in the field of immuno-oncology makes it necessary to study both disciplines simultaneously. Furthermore, these disciplines currently have enormous potential for development, and therefore the feedback of knowledge between them must be constant in order to achieve common objectives. The following is a more detailed description of fundamental aspects of immuno-oncology, which helps us to understand its relationship with nanomedicine and also might aid in finding novel applications and new actors in the field.

## 3. Immuno-Oncology

The generation of T cell-mediated anti-tumour immunity requires a series of steps that constitute a process which is called the cancer immune cycle. The understanding of the cellular and molecular mechanisms involved in these processes allows for the development of several types of immunotherapies that assist in immune activation by modulating regulatory or activating mechanisms, directing these steps to achieve an improved immune response. In contrast, cancer also employs mechanisms that delay or stop this anti-tumour immunity, called immune avoidance mechanisms. Each of these mechanisms is a part of the “cancer hallmarks” that together allow cells to acquire malignancy and then tumour development. Therefore, new approaches to improve the immune response against cancer consist of blocking these immune evasion mechanisms.

Since the cancer immune cycle was described, several strategies have been used to improve the immune processes are grouped into two types: the first one is the use of effector cells/molecules of the immune system to directly attack the tumour cells, as it is named passive immunotherapy, which includes targeted monoclonal antibodies, adoptive cell therapy, and chimeric antigen receptor-modified T cells (CAR-T). The second strategy is to improve the activation of the immune system by modulating immune regulatory mechanisms or endogenous activators, which is called active immunotherapy. In this case, different steps of the immune response can be improved, such as the absorption, processing and presentation of antigens by APCs, the activation and expansion of naive T cells or increasing the efficacious phase of the immune response. Cytokines and different types of vaccines are involved in this type of immunotherapy. Another type of active immunotherapy that is proving very successful is checkpoint inhibitors, which aim to unblock a blocked immune response to increase anti-tumour responses [73].

All of these strategies are discussed in the following sections, but first, a more thorough understanding of the “cancer hallmarks” and “cancer immune cycle” is briefly commented on, as described below.

### 3.1. Cancer Hallmarks

Tumorigenesis in humans is a multi-step process, reflecting genetic alterations that progressively lead to a continuous transformation of normal cells into highly malignant cells. Tumour genomes are altered at multiple sites, either by point mutations or by more obvious alterations, such as changes in chromosomal complement. Observations in human cancers and animal models indicate that tumour development is driven by a succession of genetic changes, which confer one or another type of growth advantage, resulting in a progressive conversion of normal cells to cancer cells. Cancer cells have defects in the signalling pathways that regulate normal cell proliferation and homeostasis. However, the cancer cells of different tumours have very broad genotype diversity. Based on this complexity, Hanahan and Weinberg proposed that these genotypes were the result of six main essential alterations: self-sufficiency in growth signals, insensitivity to growth-inhibiting signals, avoidance of programmed cell death (apoptosis), unlimited replicative potential, sustained angiogenesis, and tissue invasion and metastasis. Each of these physiological changes are capabilities acquired during tumour development that escape a cancer defence mechanism connected to cells and tissues. These six abilities are shared by most types of human tumours. These capabilities are called the “hallmarks of cancer” [74].

**Figure 4:**
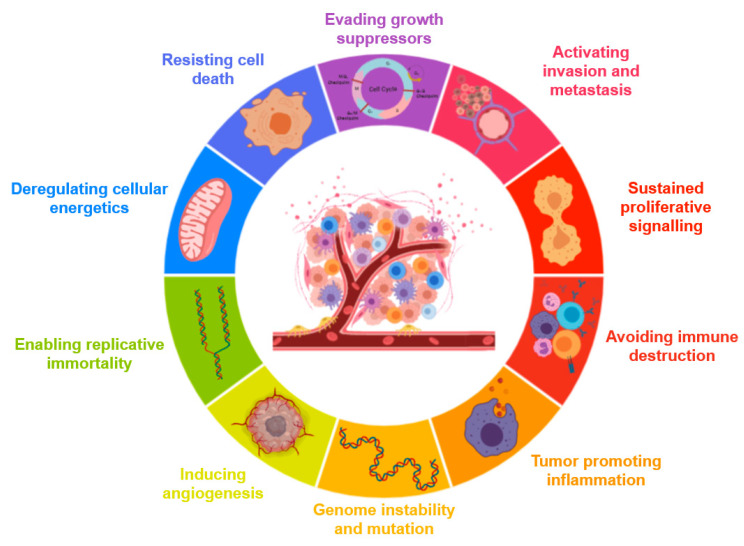
Scheme about the described hallmarks of cancer.

Later, in 2011, they determined that tumours are not just island masses of proliferating cancer cells, but are complex tissues composed of different cellular types that interact with each other. Normal cells recruited to the site of the tumour form the tumour-associated stroma and are actively involved in tumorigenesis. The biology of tumours cannot be understood by just listing the features of the cancer cells; the involvement of the tumour microenvironment must be taken into account. Four other features shared by tumours have been described: genomic instability and mutation, cellular energy dysregulation, escape from immune destruction, and tumour-promoted inflammation (Figure 4) [75]; which are also very relevant to understand the pathology to decipher therapeutically targets and also as a source for diagnostic and prognostic biomarkers.

The development of targeted therapies to treat cancer is currently very important and is based on research into the mechanisms of cancer pathogenesis. Different targeted therapies can be classified according to their effects on one or more cancer hallmarks and the efficacy of these drugs is a validation of each hallmark described.

### 3.2. Immune Cycle in Cancer

**Figure 5:**
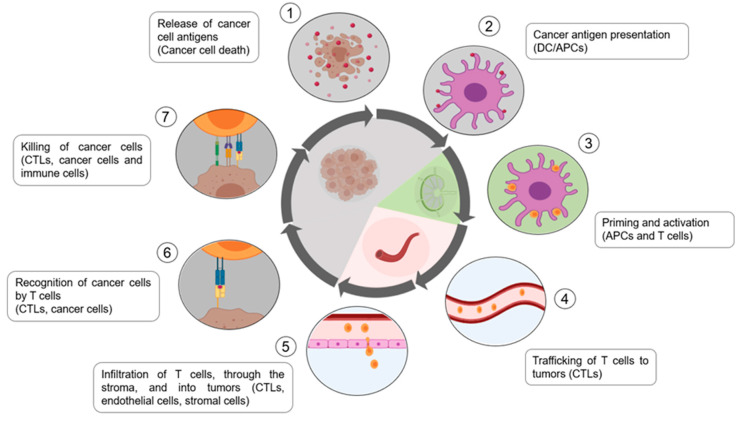
Schematic description of cancer immune cycle.

For the immune response against cancer to be effective in destroying/eliminating cancer cells, certain events must occur in a staggered and continuous manner. These events are also steps in the “cancer immune cycle” (Figure 5).

The release of neo-antigens (formed from the oncogenesis) is subsequently captured by the dendritic cells (DC) to be processed (Step 1). For this to produce an anticancer T-cell response, it must be accompanied by signals that specify immunity, thus avoiding the induction of peripheral tolerance to tumour antigens. These signals can be pro-inflammatory cytokines and factors released by damaged tumour cells. DCs then present the neoantigens on MHC-I and MHC-II molecules to T cells (step 2). Antigenic presentation on MHC molecules activates effector T cells against specific cancer antigens (step 3). It is in this step that the nature of the immune response is determined, establishing a balance between effector T cells and regulatory T cells. The effector T cells then migrate to the tumour site (step 4), infiltrating the tumour bed (step 5). Once here, the T cells specifically recognize the cancer cells and bind to them through the interaction between the T Cell Receptor (TCR) and its related antigen bound to MHC-I (step 6). Finally, the T cells kill the target cancer cell (step 7). Killing the cancer cell will release tumour-associated antigens (TAAs), causing the cycle to restart. This increases the breadth and depth of subsequent responses [76].

In cancer patients, this cycle does not work properly, with errors in the different steps described above: tumour antigens are not detected, DCs and T cells do not treat the antigens as foreign, the response is greater in regulatory T cells than in effector cells, T cells do not infiltrate tumours adequately, or even multiple factors in the tumour microenvironment may inhibit effector T cells. Bearing this in mind, the goal of cancer immunotherapy is to initiate a self-reliant cycle of cancer immunity that can amplify and spread without generating an unchecked auto-immune inflammatory response. To achieve this, immunotherapy must escape negative feedback mechanisms (checkpoints and inhibitors). Although amplifying the entire cell cycle provides anti-cancer activity, it generates damage to normal cells and tissues in return which might be drawback or source for resistance to the treatment. Recently, several clinical studies suggest that a common rate-limiting step is “immunostat function”, which is the immunosuppression that occurs in the tumour microenvironment [76].

As discussed above, different immunotherapies can act on the several phases of the cancer immune cycle to ensure that an effective immune response is generated against the tumour cells.

### 3.3. Cancer Immunotherapy

**Figure 6:**
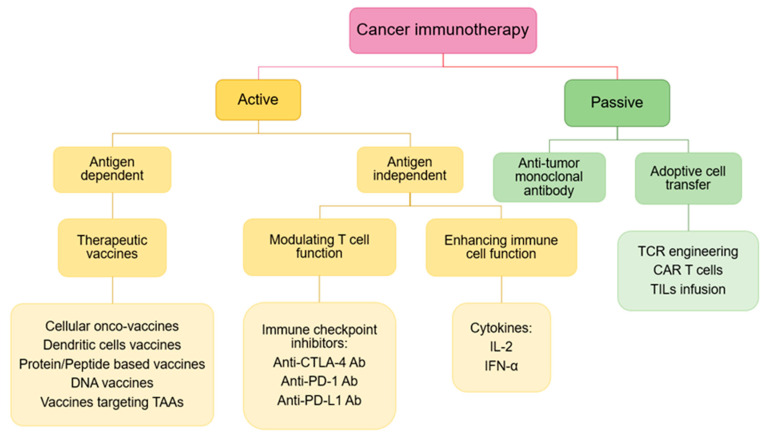
Schematic classification of immunotherapies designed for cancer.

Once the immune cycle and cancer hallmarks are described, the different immunotherapies should act and also new ones could be designed according to them. Hence, most of these immunotherapies is described briefly (from the conventional to the novel ones) (Figure 6).

#### 3.3.1. Cytokines

Cytokines are polypeptides or glycoproteins that cause growth, differentiation and inflammatory or anti-inflammatory signals to different types of cells, which are released at a particular time in response to a specific stimulus and have a limited half-life time in the circulation [77]. Target cells of cytokines express high affinity membrane receptors, which activate intracellular signalling when they bind to cytokines, producing modifications in gene transcription that will determine the cellular response. The receptors receive information about the concentration and time of exposure to different cytokines, which implies a high degree of complexity. Due to all these features, cytokines play important roles as modulating agents that are involved in immune homeostasis by regulating inflammatory response, specific immune response, tolerance mechanisms, and promoting effective pathogen control. Hence, the administration of cytokines allows for the manipulation of the immune system in auto-immune disorders, infectious diseases, increasing the efficiency of the vaccines (due to inherent adjuvants disorders) and in the therapy of cancer [78].

The ability of cytokines to enhance the immune response against cancer and the development of recombinant DNA technology has allowed for preclinical and clinical investigation of the anti-tumoral activity of several recombinant human cytokines since the 1980s [77]. Several cytokines, among others including IL-2, IL-12, IL-15, IL-21, GM-CSF and INF-α, have demonstrated efficacy in preclinical models of murine cancer [79]; however, cytokines have shown limitations, such as their short half-life and narrow therapeutic framework, with low anti-tumour efficacy in their use as monotherapy agent. So far, only a few cytokines showed clinical benefit, which were IL-2 and IFN-α, being approved by the Food and Drug Administration (FDA) as anti-tumoral therapies. In the case of IL-2, it was approved for the treatment of advanced renal cell carcinoma and metastatic melanoma; regarding IFN-α, it was approved for the treatment of hairy cell leukemia, follicular non-Hodgkin’s lymphoma, melanoma, and AIDS-related Kaposi’s sarcoma [77].

In the case of IL-2, which has been approved by FDA for the treatment of advanced renal cell carcinoma and metastatic melanoma. The identification of IL-2 as a therapeutic agent began in the 1960s, when a factor capable of stimulating lymphocyte division in antigen-activated leukocyte culture supernatants was discovered. In 1969, it was demonstrated that human lymphocyte media contained this factor and could be used to maintain T-cell cultures for more than nine months without the need for repetitive antigenic stimulation. This technique was used to cultivate tumour-reactive cytotoxic T cells. This allowed a more in-depth study of this lymphocyte growth factor, thus giving it the name IL-2 [80], which was approved for the treatment of metastatic renal cell cancer in 1992 and advanced melanoma in 1998. IL-2 has opposite functions, acting as a T-cell growth factor during the initiation of the immune response, but is also essential for terminating the T-cell response, maintaining self-tolerance. This cytokine acts as a growth factor for T CD4+ cells and NK cells and promotes the clonal expansion of antigen activated CD8 T cells. In addition, it facilitates the production of antibodies by B cells that have been previously stimulated by factors such as CD40L. With respect to its immune response attenuation function, IL-2 plays an essential role in the maintenance of peripheral Tregs cells, as well as in the Activation-Induced Cell Death (AICD) of Fas-mediated T CD4+ cells. In IACD, receptor-mediated stimulation of T CD4+ cells with high antigen concentrations induces the expression of IL-2 and their receptors, which interacts and activate the T cell cycle. This antigen activation in turn increases transcription and expression of Fas Ligand (FasL), resulting in T cell death [79].

Regarding IFN-α, it was approved for the treatment of hairy cell leukemia, follicular non-Hodgkin’s lymphoma, melanoma, and AIDS-related Kaposi’s sarcoma. IFN-α belongs to IFN type I, a family of cytokines synthesized by different cells in response to viral infections and immune stimulation [79]. IFNs of this type induce the expression of MHC class I molecules in tumour cells, involved in the maturation of DCs, activate B and T cells and increase the number of cytotoxic cells. Specifically, IFN-α has pro-apoptotic and anti-proliferative activity, but also presents anti-angiogenic activity on the tumour vasculature. The use of IFN-α was approved in 1986 for the treatment of hairy cell leukemia [77], as it produced a sustained improvement in granulocyte, platelet count and hemoglobin levels in 77% of LCH patients treated [81] and has since been used in the treatment of hematologic malignancies and solid tumours [77], such as chronic myeloid leukemia, AIDS-related Kaposi’s sarcoma, renal cell cancer, and in the case of stage II and III melanoma has been used as adjuvant therapy [79].

In contrast, administration of IL-2 and IFN-α has a low response rate and high toxicity associated with high doses, making targeted therapy and checkpoint inhibitors a better option currently for these tumours [77].

A drawback of treatments with cytokines is that, for some of them, positive actions are accompanied by the induction of immune checkpoint cytokines, such as the inhibitory factors IL-10 or TGFβ [79]. IL-10 is released by innate and adaptive immune cells to regulate the activity of pro-inflammatory cytokines; but also as an immunosuppressive cytokine, because it decreases the antigen-presenting activity of dendritic cells (DCs) and inhibits cytotoxic function and cytokine release from T and NK cells (depending on the microenvironment). In chronic infections and cancer, CD8+ T cells exhibit autocrine activity mediated by IL-10, inhibiting their antigen-induced apoptosis, thus prolonging the efficacious activity of cytotoxic lymphocytes. TGFβ has a dual role in the tumour process, since at the beginning of tumorigenesis, TGFβ is an inhibitor of tumour development by blocking the cell cycle; nevertheless, in later stages, the cells develop mechanisms of resistance against the TGFβ´s effects. This resistance mechanism begins to promote tumour progression and mediates the epithelium-mesenchyme transition. In addition, TGFβ promotes the release of angiogenic factors (such as vascular endothelial growth factor (VEGF)), and the recruitment of Treg cells, neutrophils, macrophages (with pro-tumour polarization), myeloid-derived suppressor cells (MDSC) and tolerogenic DCs, in turn decreasing the functions of NK cells and CD8 T lymphocytes [77].

In summary, cytokines have demonstrated anti-tumour therapeutic activity in murine models and in the clinical treatment of certain specific human cancers. Moreover, IL-2 and IFN-α have been approved for the treatment of selected malignancies. In contrast, cytokines in monotherapy have not met all the expectations efficiency as has been observed in preclinical experiments. This is because they are often associated with severe dose-limiting toxicities, and are known to induce immunosuppressive humoral factors, suppressive cells and immune checkpoints. Normally, soluble cytokines act over short distances, in a paracrine or autocrine manner; therefore, to achieve effective intra-tumoral concentrations they must be administered parenterally at high doses, which increases the potential for systemic toxicities, such as hypotension, acute renal failure, respiratory failure and neuropsychiatric symptoms in severe situations. They also do not induce a tumour-specific immune response. To avoid these drawbacks, new mutant engineered cytokines (supercins), chimeric antibody-cytokine fusion proteins (immunocins) or even the combination of cytokines with other therapies such as checkpoint inhibitors, among other novel strategies, are being investigated in an attempt to increase their anticancer efficacy [82]. However, due to these limitations, it has been necessary to develop more tumour-specific immunotherapeutic agents with greater effectiveness and less associated toxicity that are currently being used with better results, and the employment of cytokines in immunotherapy has taken a back seat.

#### 3.3.2. Monoclonal Antibodies

The first monoclonal antibodies (mAb) to be clinically tested as a cancer treatment were murine mABs, but their problems of administration in humans limited their clinical usefulness [83]. The success of mAbs therapy came with the development of techniques that allowed the genetic modification of murine mAb to produce murine–human chimeric mAb or humanized mAb, which behaves like human IgG.

These antibodies have some advantages, such as their specific binding to molecular epitopes, interaction with the effector arms of the immune system, their long half-life, the ability to distribute themselves in the intra- and extravascular compartments and that the host tolerates IgGs well as therapeutic agents. In addition, they can be produced in large quantities and at a controlled cost. Due to their effective bio-distribution, systemic mAbs levels last for weeks or months, mediating a prolonged anti-cancer response. mAb can attack tumour cells by binding to tumour-associated antigens (TAAs) and modifying signalling or directing immune effector mechanisms to those tumour cells [84].

There is currently a wide diversity of mAb-based strategies for cancer therapy. The optimal characteristics for a targeted tumour antigen depend on the mAb to be used, the nature of the tumour and the mechanism of action of these mAb.

mAbs that target cell surface antigens can induce apoptosis by direct transmembrane signalling, by complement-mediated cytotoxicity or by inducing antibody-dependent cell cytotoxicity [85,86]. Determining the most appropriate mechanism for each mAb depends on the clinical scenario and is a continuous scientific challenge.

mAb could induce tumour cell death by target cell signalling. However, resistance can arise when cells with alternative or compensatory signalling pathways appear. The use of combination therapy may overcome these resistances. An example is mAbs against the ErbB family of receptors and their ligands, such as Trastuzumab and Pertuzumab [87,88]. The mechanism of these mAbs is complex, as the receptors can have multiple ligands and mAbs can alter the dimerization properties, interfering in different signalling depending on whether it is directed to a homodimer or heterodimer receptor [84].

For mAbs measuring complement-mediated cytotoxicity (CMC), it is known that their ability to bind complement and induce CMC depends on the antigen concentration, membrane orientation and whether the antigen is in monomer or polymer form. CMC also depends on the mAb isotype and the characteristics of the target cell. Some of these mAbs are anti-CD20, in chronic lymphocytic leukemia (CLL), such as rituximab or obinutuzumab. CMC contributes most to the effect of mAb in hematological malignancies, where target cells are exposed to complement system in the circulation [89].

mAbs can also induce antibody-dependent cell cytotoxicity (ADCC), mediated by FcR binding, which is expressed by immune effecting cells such as NK, granulocytes and monocytes/macrophages [90,91]. The mAb binds to the target cell through FcR, which activates intracellular signals through immunoreceptor tyrosine-based activation motifs (ITAM) and induces the activation of the effector cell, thus producing ADCC.

Many of the tumour associated antigens (TAAs) are not expressed on the surface of the tumour cells but are presented by MHC molecules. Therefore, mAbs have been developed that recognize these peptides, which come from intracellular oncoproteins. These antibodies are restricted by MHC and are still under development and further characterisation [84].

Molecule-specific mAbs that have an impact on the host can block tumour angiogenesis, preventing tumour growth, or target immune checkpoints, enhancing the anti-tumour immune response. In the first case, the mAb that blocks angiogenesis is bevacizumab, which blocks vascular endothelial growth factor (VEGF). This has an anti-tumour effect, as it prevents the passage of nutrients and oxygen to the tumour [92]. As these mAbs do not directly target the tumour, they are usually combined with cytotoxic agents [93]. Bevacizumab is effective in colorectal, lung, breast, renal, brain and ovarian cancer. The mAbs targeting immune checkpoints are described in a following section.

Antibody-drug immunoconjugates and radio-immunoconjugates that deliver a toxic load to tumour cells may also be used. Bi-functional antibodies and Chimeric Antigen Receptor T cells (CAR-T cells) can take advantage of the specificity of mAb to guide the cellular immune system to tumour cells [84]. Therefore, improved mAb-based therapeutic agents are being developed with multiple possibilities in cancer immunotherapy.

#### 3.3.3. CAR-T Cells

This modality of immunotherapy is one of the newest adoptive cell therapy (ACT) strategies in cancer treatment. However, before knowing why it has such an impact as a potential cancer immunotherapy treatment, it is necessary to describe how it has been developed from the first ACT attempts.

**Figure 7:**
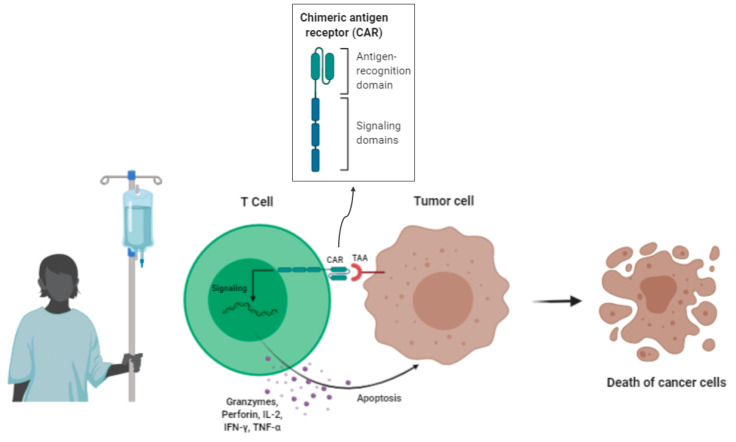
Schematic description of chimeric antigen receptor-modified T cells (CAR-T) cell therapy: structure and mechanism of action.

Based on the idea that tumour-specific T cells could eliminate tumour cells, ACT was developed, which involves the therapeutic use of T cells, passively administrated (Figure 7) [94].

ACT has some advantages over other approaches to cancer immunotherapy. Large numbers of anti-tumour T cells can be grown in vitro and selected for their high avidity against the desired antigen. In addition, the host can be manipulated prior to administration of these cells to provide a suitable microenvironment in the tumour [95].

Following the use of IL-2 as a T-cell growth factor in the treatment of patients with metastatic melanoma and renal cell cancer (RCC), manipulation of the host immune system has been suggested to elicit an endogenous reaction capable of mediating cancer regression. The most potent cells were tumour infiltrating lymphocytes (TIL) grown from tumour fragments [96]. The first use of TILs was performed by the Surgery Branch, National Cancer Institute (NCI) in 1988 in the treatment of patients with metastatic melanoma [97]. Several TIL studies have shown that cells with anti-tumour activity can be isolated from tumours derived from patients with melanoma, but in most other tumour types these cells are difficult to isolate and spread and do not recognise tumour antigens. Therefore, techniques were developed to introduce anti-tumour T cell receptors (TCR) into autologous lymphocytes for use in therapy. Conventional TCRs αβ and chimeric antigen receptors (CAR) with anti-tumour specificity can be introduced into normal lymphocytes, providing them with anti-tumour activity. The redirection of T-cell specificity with conventional TCR αβ receptors is HLA-restricted, limiting treatment to patients expressing a particular HLA haplotype. TCRs, on the other hand, are not restricted to HLA, but are limited by the need for expression of the tumour antigen on the cell surface. In addition, CAR can also recognize carbohydrate and lipid debris, which has greater potential application [95].

Therefore, the use of Chimeric Antigen Receptor modified T cells (CAR-T cells) attempt to combine the high affinity of antibody fragments targeting tumour antigens with the destructive function of T lymphocytes [94].

Essentially, CAR-T cells are synthetic constructions that bind to target cell surface antigens using a single-chain variable fragment recognition (scFv) domain. The first designed generation of CAR-T cells consists of a scFv domain linked to a 3-zeta-strand differentiation cluster (CD3ζ) that induces the activation of T cells after binding to the antigen. This CD3ζ chain can only deliver a single strong intracellular signal (as it does not contain the chains γ, δ and ε that normally make up the TCR-CD3 complex which are required to amplify intracellular signal. In order to improve the CAR molecule, the second and third generation of these CAR-T cells were developed, incorporating other intracellular signalling domains such as CD28, CD137 and ICOS (inducible T cell co-stimulator). Cytokine receptor signalling or inflammatory cytokine expression domains such as IL-12 or IL-18 have been included in fourth and fifth generation CAR-T cells [94].

CAR-T cell therapies have been successful in several hematological malignancies but are less effective in treating most solid tumours. Since 2010, multiple CAR-T cell clinical trials have been conducted targeting CD19 (CD19-CAR-T cells) to promote clinical responses in acute lymphoblastic leukemia (ALL) [98,99], diffuse large B-cell lymphoma (DLBCL) [100], chronic lymphocytic leukemia (CLL) [101], and other non-Hodgkin’s B-cell lymphomas [102], with remissions of up to 90% in some cases. This is because CD19 is always expressed in the B cell lineage and attacking CD19 eliminates this cell compartment in patients. Although this advantage may also appear to be a disadvantage, B cell aplasia can be treated with immunoglobulins and is therefore a manageable toxicity [103].

Two constructs of CD19-CAR-T cells have been approved by the FDA for their excellent results in refractory patients to standard therapies. They are Tisagenlecleucel (co-stimulatory domain 4-1BB/CD3ζ), approved in 2017 for B-ALL and in 2018 for DLBCL; and axicabtagene ciloleucel (co-stimulatory domain CD28/ CD3ζ), approved in 2017 for DLBCL. These approvals make CAR-T cells the first FDA-approved personalised gene therapy [104].

In malignant CD19+ refractory B-cell tumours, CD19-CAR-T cells have been shown to be clinically effective. However, these studies have also shown that relapse of the disease is more frequent in antigen-negative tumours, so it is important to determine the loss of antigen for these therapies [94].

On the other hand, monitoring the toxicity of the CAR-T cells is also important. The toxicity associated with this therapy is mainly outside the tumour, which is an obstacle in the clinical development of these therapies, and therefore, it is also very important to select the targets appropriately. The toxicity associated with CAR-T cells must be reversible after the elimination of the target cells or after the exhaustion of the T cells [94].

One of the bottlenecks is that T-lymphocytes are required to be removed from patients’ peripheral blood and amplified in vitro, which is complex and time-consuming. To overcome these limitations, the in-situ construction of CAR-T in vivo seems to be the best option. Here, nanomedicine could help to improve the potential of these treatments and overcome mostly of the drawbacks. One of the approaches recently described is based on NPs coated with poly-β-amino-ester with reversible bound plasmid DNA encoding leukemia-specific CAR, which are internalised in the lymphocytes by anti-CD3 antibody-mediated endocytosis. Subsequently, the NPs selectively transfected with CAR genes into the nuclei of the patient’s T cells. The T cells programmed by the synthetic NPs were found to in vitro express CAR after 24–48h incubation period. After in vivo administration, the NPs were identified and rapidly bound to the peripheral circulating T cells (abundant in the spleen, lymph nodes and bone marrow of the mice), showing an increase in overall survival rate. Despite the above, it has not yet been verified whether this methodology can effectively produce CAR-T cells and a long-lasting immune response in the human body, as well as whether toxicity problems can occur due to possible off-target effects [46].

Although this success of CAR-T cells has not yet been achieved in patients with solid tumours, the development of CAR-T cells in these solid tumours is still in its early stages. In solid tumours, the first obstacle is to design a CAR-T against an antigen that is expressed in the tumour but not in the normal tissue. Due to this difficulty, CAR-T cells in these tumours have presented serious toxicities until now. Although some tumour specific antigens have been identified, CAR-T cells have had very low efficacy against these target antigens in the clinic [104]. In the case of solid tumours, the effects outside of the tumour could lead to widespread cytokine release, resulting in organ failure. In order to exploit unique neo-antigens in solid tumours, their specific surface accessible expression would be required and combined with the production of immunoglobulins or nano-antibodies (HHV) would have to recognise them in order to generate specific CAR-T cells [105]. In addition, if a perfect antigen is found in solid tumours, CAR-T cell therapies in these types of tumours have to deal with other problems, such as poor traffic to the tumour site or limited persistence and proliferation within the host. The TME of these tumours may also functionally suppress CAR-T cells [104].

Therefore, it could be useful to compromise the microenvironment of solid tumours to delay their growth. The TME of many solid tumours share some characteristics, such as the expression of inhibitory molecules like PD-L1. Hence, a CAR-T cell that recognizes PD-L1 should palliate immune inhibition and allow for the activation of CAR-T cells in the TME, dampening immunosuppressive signals and promoting inflammation [105].

In the solid tumours, the suppressive TME inactivates TILs through the production of immunosuppressive molecules, and inflammatory cytokines are released from the treatment itself (IFN-γ, TNF-α), which is attributed to systemic administration. Targeted therapy based on NPs is required to remodel TME without causing systemic toxicity [46].

Solid tumours depend on the extracellular matrix (ECM) and the neo-vasculature for nutrient supply, which may be another target for T-CAR cells since tumour ECM and new blood vessels have unique antigens that are not present in healthy adults. Based on this, the group led by Yushu Joy Xie has designed a CAR-T cell which can be generated using an HHV that recognizes EIIIB, which is a splice variant of fibronectin that is expressed in a high form in tumoral ECM and neo-vasculature. This may improve the local inflammatory response and drug access to the tumour in otherwise impervious cancers [105].

Both CAR-Ts that recognize PD-L1 and those that recognize EIIIB have been tested in a B16 melanoma model and have shown significant delay in tumour growth and improved survival in both cases [105].

In summary, ACT with CAR-redirected T cells is a potentially curative strategy in patients with tumours resistant to standard treatments. CAR-T cells have demonstrated their potency in hematologic cancers, as reflected by their FDA approval for B-ALL and DLBCL. On the other hand, for solid tumours, this therapy is still in an early stage of development and may require a new approach to improve its effectiveness.

#### 3.3.4. Therapeutic Onco-Vaccines

Another therapeutic strategy is onco-vaccines. Onco-vaccines represent one of the viable options for active immunotherapy against cancer by using the patient’s own immune system. Different to prophylactic vaccines, which are administered to healthy individuals, therapeutic vaccines are administered to cancer patients with the aim of eradicating the cancer cells [106].

**Figure 8:**
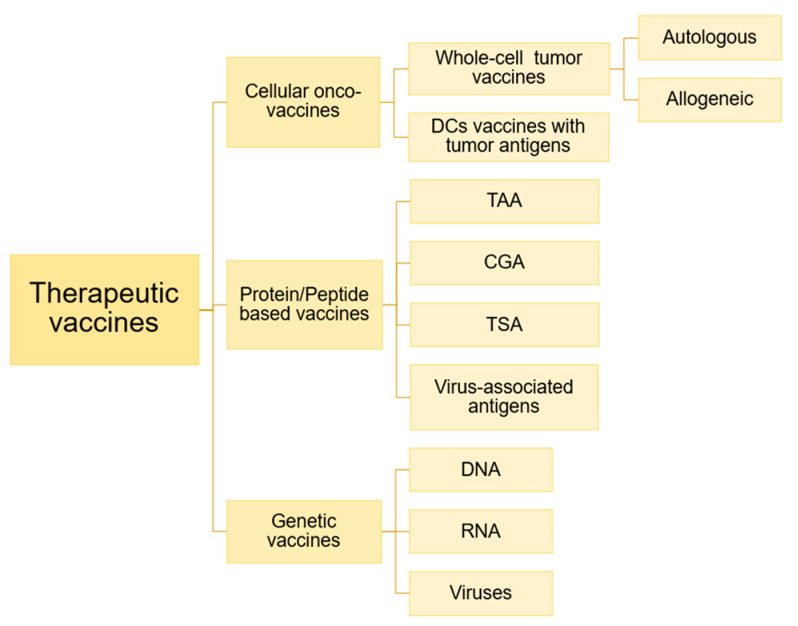
Schematic classification of currently available onco-vaccines.

In general, onco-vaccines are classified depending on their format/content: cellular vaccines, protein/peptide vaccines and genetic vaccines (DNA, RNA and viruses) (Figure 8) [106,107].

**Figure 9:**
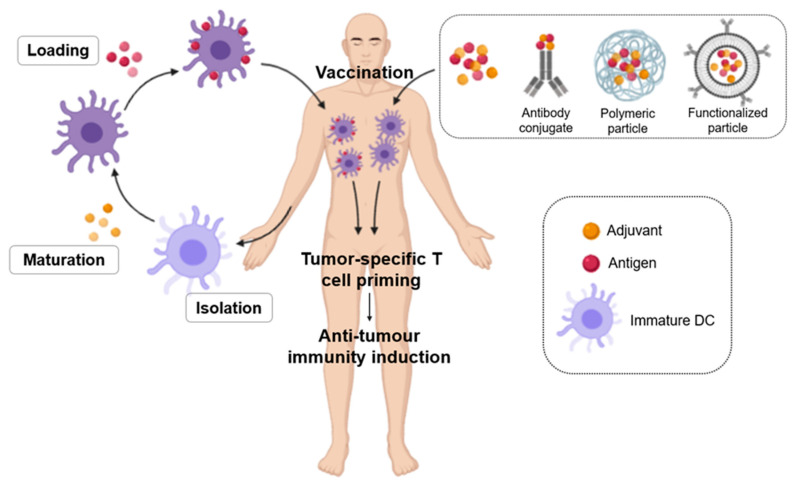
Inducing anti-tumour immune responses by dendritic cell (DC) vaccination through infusing patients with ex vivo antigen loaded DCs (left) or targeting antigens and adjuvants directly to DCs in vivo (right).

The main characteristics of each group are:

1. *i. Cellular onco-vaccines*: Within cell-based vaccines there are two types: (i) autologous or allogeneic whole-cell tumour vaccines and (ii) autologous dendritic cells, pulsed or transfected with tumour antigens (contained in tumour lysates, purified proteins, peptides, DNA or RNA) [108]. Autologous cell-based vaccines are based on patient-derived tumour cells, which are irradiated and combined with an immunostimulatory adjuvant and administered to the same individual from whom the cells were extracted and isolated [109]. These vaccines have been tested in a variety of solid cancers, including lung cancer, colorectal cancer, melanoma, renal cell cancer, and prostate cancer [106], showing potent antitumour immunity in preclinical animal models and, in early human clinical trials, have shown relative safety, as well as the induction of tumour-specific immune responses and evidence of antitumour activity, obtaining clinical benefit, although objective response rates remain low [110,111,112,113]. One of the advantages of this type of vaccine is that it has a high potential to deliver the full spectrum of Tumour-Associated Antigens (TAAs) and, in addition, autologous tumour cells can be modified to acquire more potent immunostimulatory characteristics [106]. However, there are some disadvantages, such as requiring an enough tumour sample and potentially inducing autoimmunity, as tumours also express patient-specific proteins [114]. Allogeneic tumour cell vaccines typically contain two or three human tumour cell lines, and have the advantage that they contain unlimited sources of tumour antigens and can produce standardized, large-scale vaccines [106]. An example is Canvaxin, which contains three melanoma lines combined with Bacillus Calmatte-Guerin (BCG) as an adjuvant [115]. In 2010, the first cell-based vaccine was approved by the FDA, based on dendritic cell vaccine called provenge (sipuleucel-T), which targets Prostatic Acid Phosphatase (PAP) antigen in castration-resistant metastatic prostate cancer. PAP is an TAA, which gives the vaccine some specificity and therefore improves the anti-cancer effect [116]. Other vaccines that use whole tumour cells as antigens are OncoVAX for colon cancer and GVAX for prostate cancer [117,118]. These cells can also be genetically modified to produce immune molecules, as in the case of Lucanix for NSCLC [119]. The disadvantage of cell-based vaccines is that they are expensive and, in the case of autologous vaccines, it is difficult to produce them on a large scale [107].
2. *ii. Dendritic Cell (DC) Vaccines*: These vaccines are based on the main characteristic of DCs, which are professional antigen-presenting cells. DCs act in the peripheral tissues, where they absorb, process and present antigenic peptides of the pathogen or host to the virgin T lymphocytes in the lymphoid organs through the MHC. Therefore, DCs are important for connecting innate and adaptive immunity. Functional characterisation in DCs determine that three signals are necessary for complete activation of DCs: 1. adequate loading of MHC–peptide complexes in DC for priming of T cells; 2. positive regulation of co-stimulatory molecules such as CD40, CD80 and CD86, 3. production of cytokines that polarize the Th1/Tc1 immune response [106]. Ex vivo generated DCs are used as cancer vaccines. For this purpose, human DCs can be generated in culture from CD34+ hematopoietic progenitors or peripheral blood monocytes [120]. DC vaccines are achieved by loading TAAs antigens on autologous DCs from patients, which are then treated with adjuvants (Figure 9) [106]. For example, GM-CSF is essential for ex vivo generation of monocyte-derived DC [121]. These cells required a maturation process, which is associated with morphological and functional changes in the DC, allowing improved expression of MHC-I and -II, co-stimulatory molecules and increased cytokine production [122]. These ex vivo DCs are then administered to patients to induce anti-tumour immunity. Thus, T cell activation is regulated by co-stimulatory molecules expressed in DC, so the potency of the DC vaccine can be improved by modifying the expression levels of these inhibitory or activating molecules. DCs need stimulation of CD40 by active CD4+ T cells, so human DCs expressing high CD40L lead to increased activation of reactive T cells with low immunogenic tumour antigens. The activating molecules expressed in DC are related to the response of pro-inflammatory T cells, while suppressor molecules contribute to the tolerance or suppression of T cells [106]. The first work that laid the foundation for DC vaccine development was carried out by Inaba et al. in 1992. They cultivated mouse DC ex vivo from bone marrow precursors [123]. One of the first trials testing the immunogenicity of DC was performed on metastatic prostate cancer. Patients received autologous pulsed DCs with peptides restricted to HLA-A0201 derived from the prostate-specific membrane antigen (PSMA). Antigen-specific cellular responses and reduced PSA levels were observed in some patients [124]. These vaccines have also been tested in clinical trials for the treatment of prostate cancer, melanoma, renal cell carcinoma, and glioma [125,126,127,128,129,130,131]. The results of these studies are mixed but ultimately indicate that, although studies in mice demonstrate a potent ability of DCs to induce antitumour immunity and autologous DCs generated from peripheral blood in humans are a safe and promising approach, further studies are still needed to demonstrate their clinical efficacy and impact on the survival of patients with these types of cancers. As mentioned above, the DC vaccine Sipuleucel-T (Provenge TM) is the first therapeutic cancer vaccine approved by the FDA and has succeeded in increasing survival with a favourable toxicity profile, opening up new paradigms in cancer treatment [106].
  *iii. Protein or peptide-based vaccines*: These vaccines are based on tumour-associated antigens (TAA), cancer germline antigens (CGA), virus-associated antigens or tumour-specific antigens (TSA), along with some adjuvants. Those composed of synthetic peptides generally contain between 20 and 30 amino acids directed at specific epitopes of tumour antigens. Antigens can be modified to bind cytokines, antibodies or immunogenic peptides in these vaccines [107]. In this group of vaccines, a few representative examples are Oncophage, which is used in kidney cancer, melanoma, and brain cancer; and MUC1, which is used in breast cancer and NSCLC [132,133]. These types of vaccines are not very expensive and are also very stable but have the limitation that known peptide epitopes are required to be candidates for use in vaccines. Other disadvantages are immune suppression and the weak immunogenicity of these antigens [134]. Recombinant vaccines based on TAA peptides are classified into different categories: 1. antigens encoded by genes that are normally silenced in adult tissues, but which are transcriptionally reactivated in tumour cells (testicular cancer antigens, such as melanoma associated antigen (MAGE) and SSX-2), 2. Tissue-differentiating antigens, which have a normal tissue origin and appear in both normal and tumour tissue (melanoma, breast carcinomas and prostate cancer, such as gp100, mammaglobin-A and PSA, respectively), 3. Tissue differentiation antigens similar to the above, but which, compared to their normal homologous tissues, are very high in tumour tissues (MUC-1, HER2, p53, hTERT, etc.), 4. tumour-specific antigens, which are normally mutated oncogenes (e.g., Ras, B-Raf) and 5. molecules associated with tumour stem cells or with the epithelium-mesenchyme transition process [106]. This type of vaccine is more cost-effective than individualized vaccines, but also has the disadvantage of targeting only one or a few epitopes of the TAAs. To improve the immunogenicity of an auto-antigen, the peptide sequence of TAAs can be altered by introducing agonist-enhancing epitopes that increase peptide binding to MHC or TCR, enhancing the T cell response against the target [106]. Immuno-stimulatory adjuvants are also used when the TAA display of a weak immunogenic nature. Aluminium salts have been used as adjuvants to promote humoral immunity but are not effective in diseases requiring cellular immunity. To induce the adaptive immune response, activation of innate immunity is necessary, which has led to questions about theories of how adjuvants promote adaptive immunity [106]. Charles Janeway demonstrated that adaptive immune responses are dependent on innate immune receptors activated by microbial components [135]. Pattern-Associated Molecular Pattern Recognition (PAMPs) through pattern recognition receptors (PRRs) involves the coordination of innate and adaptive immunity to microbial pathogens or infected cells. TLR-mediated activation of DC is very important in this process, which is why many vaccines include PAMPs as part of therapeutic immunizations against cancer. That is, these molecules are used as adjuvants, facilitating the development of vaccines. Some examples are the use of BCG to treat bladder carcinoma, by activating TLR2 and TLR4, or LPS, which is a natural ligand of TLR4 [106].
3. *iv. DNA Vaccines:* These are vaccines in the form of genes use either DNA, such as plasmids, or RNA, such as mRNA [107]. Viral DNA vectors can transfuse infiltrated somatic cells or DCs as part of the inflammatory response to vaccination [106]. APCs absorb genetic material and translate peptide and proteins as cancer-specific antigens, stimulating the immune response [107]. Currently, there are some DNA vaccines include mammaglobin-A for breast cancer, PAP for prostate cancer, and gp100 and gp75 DNA for melanoma [136,137,138,139]. Disadvantages may be the method of DNA/RNA delivery and the efficiency of absorption, which may limit transcription and antigenic presentation by APCs [107]. These vaccines have been administered using viral vectors and electroporation, which are effective but difficult to apply in the clinical routine [140,141]. It should also be noted that the administration of live virus may cause side effects and decrease the effectiveness of antiviral antibodies in patients [140].
4. *v. Vaccines targeting TAAs*: To achieve tumour-specific death, cancer vaccines must target restricted epitopes of MHC-I that activate CD8+ T cells, as these are the most potent cells and when activated recognize TSAs and distinguish normal cells from cancer cells [142]. This involves the following processes: degradation of ubiquitous proteins by the proteasome, interaction of peptides with Hsp90 in the cytosol, which acts as a chaperone, active transport into the endoplasmic reticulum by the TAP transporter, modification of peptides by ERAP to an appropriate length, which are subsequently loaded into the peptide-binding cleft of MHC class I molecules with the help of chaperones such as tapain and transport to the cell surface, and can thus be recognised by the CD8+ T-cell receptor [143]. There are different types of tumour antigens that can be targeted in immunotherapy: (i) tumour-associated antigens (TAA), which are over-expressed on tumour cells and are expressed to a lesser extent on normal cells, (ii) cancer germ-line antigens (CGA), which on normal adult cells are found only in reproductive tissues, but are expressed selectively on several types of tumours, (iii) virus-associated antigens, which arise in tumour cells from oncogenic viral proteins; and (iv) tumour-specific antigens (TSAs), which are the neo-antigens and are only found in tumour cells, as they arise from non-anonymous somatic mutations [107]. Commonly, cancer vaccines should target the broadest possible antigen repertoire, which can be achieved by using autologous tumour lysates, whole-tumour-derived mRNA, irradiated autologous tumour cells, or allogeneic tumour cell lines [144,145]. In addition, effective responses in response to an antigen can result in the immunogenic release of additional endogenous antigens by tumour cell destruction, leading to a broader immune response. This is known as “epitope spread” [146]. Vaccines targeting TAAs have not been very successful so far and are still under development, mainly because many TAAs are also expressed on normal cells, which show central and peripheral tolerance, and the affinity of TCR for these antigens might be very low [147]. In addition, autoimmune toxicities may take place during treatment. Despite this, some AATs are used as targets Despite the weak points on this approach; Currently, several approaches has been quite promising and help to open more studies exploring the full potential, for example: CD19-directed CAR-T therapy in acute lymphoblastic leukemia (ALL), which results in complete remission in a large number of patients [148]. CGAs, such as melanoma associated antigen 3 (MAGE-A3) and NY-ESO-1 antigen, are expressed selectively in some cancers, but when used as a target they result in high toxicities. In particular, severe neurological toxicities and death occur when MAGE-A3 is targeted [149]. On the other hand, virus-coded antigens are only present on tumour cells, not on normal cells, as some cancers are associated with virus infection. Viral oncogenes encode oncoproteins that cause cell transformation. An example is the human papilloma virus (HPV), which is associated with cervical cancer [150]. This method has been effective in treating cancer, but there are also virus-associated antigens with the ability to escape from the immune system [151]. In the approach of these vaccines, the critical and important key aspect is the selection of tumour-specific antigens (TSA), which are the neo-antigens. These are peptides that arise from non-anonymous mutations, alterations in genomic codons, editing, processing and antigen presentation in tumour cells [107]. Among all non-synonymous mutations, a part of them is distributed clonally by the tumour and generates peptides containing mutations (neo-epitopes) that can be recognised by cytotoxic T cells. Deletions and insertions are also highly predictive of response [121]. The use of these mutant derived epitopes is based initially on the responses to checkpoint inhibitors, which are proportional to the mutational load of each tumour [152]. Neoantigens are presented by MHC on the cell surface in order to be recognised by the T lymphocytes of the immune system. TSAs are the best therapeutic targets for cancer vaccines and T-cell-based immunotherapy because they are different from the germline and are not considered proprietary by the immune system. In addition, they are not subject to central or peripheral tolerance, as normal cells do not express them, so they will not cause auto-immunity problems either [107]. To identify immunogenic neo-epitopes in each patient, a combination of genomic sequencing of the tumour, RNA sequencing and bioinformatic tools with algorithms that allow for the prediction of the mutations are required, which will be presented to the T cells based on the processing by the proteasome and the affinity of the molecules for human leukocyte antigen (HLA). The resulting sequences can be synthesized as mRNA or as peptides for use as a vaccine. This methodology has been validated in preclinical trials, demonstrating that mutanome-derived neoantigens can induce an immune response against autologous tumours [153]. There are also phase 1 trials showing the immunogenicity and viability of the vaccine against the neo-antigen in metastatic melanoma [154]. The disadvantage of this customized approach is that it is a lengthy process and is therefore only suitable for certain patients. Neo-antigens have already been identified in different types of cancer such as melanoma, lung cancer, liver and renal cancer [155]. Adoptive cell transfer (ACT) studies of autologous tumour infiltrating lymphocytes (TIL) have shown that an effective antitumour immune response occurs in the presence of tumour specific T cells [156]. Isolated T cell clones or TCR-designed T lymphocytes have demonstrated the epitope patterns of neoantigens that are recognised by T cells [157]. Increasingly, cancer vaccines are being designed based on neo-antigens, targeting immunogenic mutations unique to each patient. Customized RNA mutanome vaccines and peptide-based vaccines have been tested and found to be safe and capable of eliciting T cell responses to neo-epitopes in melanoma patients [154,158]. When neo-epitopes are presented by antigen-presenting cells (APCs), such as dendritic cells and tumour cells themselves, cross presentation—whereby antigen-presenting cells phagocytize exogenous antigens and process them for presentation by MHC-I—plays an important role [159]. For a sufficient response of T cells to a neo-epitope, it is important to consider the affinity of the TCR for its related antigen [142]. Because neo-antigens are small pieces of peptides that contain tumour mutations, immunization with these antigens requires the assistance of other immune-stimulatory agents to produce an efficient immune response. On their own, peptides as vaccines may not be able to stimulate the immune system in a potent way, so they are used in combination with adjuvants [160]. Generally, to activate cytotoxic T cells and obtain a potent immune response, the stimulation of T helper cells is also required [142]. Even peptides with epitopes capable of activating cytotoxic T cells and helper T cells need an adjuvant to obtain an effective vaccine, so containing a potent immune-stimulator is very important to obtain an effective response. Then, CD8+ T cells are induced [161]. The appropriate adjuvant must be able to induce the production of cytokines and co-stimulator molecules from APC and also be able to deliver the optimal amount of antigen, to maintain a balance between antigen persistence, antigen concentration and antigen distribution [162]. In addition, the adjuvant must enhance cell-mediated immunity polarized to type 1 [121]. Adjuvants can function in several ways: gradually releasing the antigen, stimulating pattern recognition receptors in APCs, protecting antigens from rapid degradation, and extending antigen presentation time [142]. Different types of cells with neo-epitopes have also been pressed for immunization, such as B cells, macrophages, splenocytes or dendritic cells, which serve as delivery and adjuvant systems [142]. Since dendritic cells are capable of efficiently capturing, processing and presenting the antigen, initiating the immune response, they are also considered natural adjuvants, but the number of dendritic cells presented in peripheral blood in cancer patients is very low, in addition, this DCs may not be functional due to the effect of TME, so one of the goals is to provide enough functional DCs for each patient. It is also important to determine the DC subtype that works best as an adjuvant, the number of DCs injected, their stage of maturation or the location of the injection [163].
5. The identification of neo-epitopes is the most specific approach to cancer treatment, since it allows for a targeted immune response against specific tumour epitopes, but with this approach, no clinically determinant results have been achieved, since these strategies are conditioned by the TME, T-cell depletion, regulation of the immune checkpoint, tumour heterogeneity, etc. For this reason, it is necessary to find an ideal combination of neo-epitope vaccines, chemotherapy, radiotherapy, checkpoint blocking therapies, etc., specific to each patient [142].

#### 3.3.5. Checkpoints Inhibitors

T cells play a critical role in the recognition together with the effector cells of the acquired immune response, and their activation requires the presence of two signals: the antigen-specific signal, mediated by TCR and MHC, and the co-stimulatory signal, mediated by membrane protein molecules expressed on the surface of the T cells and their ligands. The co-stimulatory molecules of the T cell activation signals enhance the immune responses mediated by TCR signalling. These molecules initiate, stimulate, amplify and enhance the immune response at different stages, also controlling its extension and duration. In tumour tissues, negative regulatory checkpoints predominate, inhibiting T cell activation, thus allowing tumour cells to evade the immune response and generating an immune tolerance of the tumour. Therefore, immune checkpoints (ICs) are key to maintaining self-tolerance, protecting the body against autoimmunity and inflammation by interfering with the cytotoxic T cell (CTL) immune response. Pathways that inhibit the immune checkpoint are always activated in inflammatory MSDs, allowing tumour cells to evade immune surveillance, also eradicating the immune response of TILs. Different types of Immune Checkpoint Inhibitors (ICIs) have been developed to reactivate these dysfunctional T cells [46].

**Figure 10:**
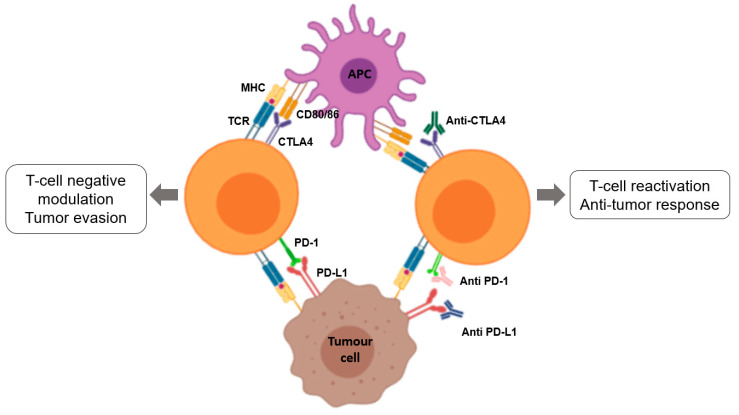
Schematic representation of the mechanisms of immune-checkpoint inhibitors (ICIs).

Checkpoint inhibitors are monoclonal antibodies that block CTLA-4 (Cytotoxic T-Lymphocyte-associated Antigen 4), PD-1 (Programmed cell Death receptor) or its ligand PD-L1 (Figure 10) [77].

• CTLA-4:

CTLA-4 is a leukocyte differentiation antigen and a transmembrane receptor on T cells, which shares the B7 ligand with its co-stimulator molecule receptor (CD28). When CTLA-4 binds to B7 it stimulates T-cell anergy, i.e., it participates in the negative regulation of the immune response by inducing a lack of T-cell response and preventing T-cell activation. The antibody to CTLA-4 has the following anti-tumour mechanisms: (1) modulation of tumour-specific immune effector cells, such as CD8+ T cells, to promote their clonal proliferation, (2) removal of Tregs to reduce inhibition of tumour-associated immune response [46].

• PD1/PD-L1:

The PD-1 and PD-L1 checkpoints limit the excessive immune response to antigens and prevent autoimmunity. PD-1 is expressed in different immune cells such as NK cells, B-lymphocytes, T-lymphocytes, DC and activated monocytes. PD-L1 is overexpressed on tumour cells and promotes cancer avoidance of immune surveillance by inhibiting CTLs. The PD-1/PD-L1 pathway modulates immunosuppression by the following mechanisms: (1) the binding of PD-L1 on the surface of tumour cells and myeloid-derived suppressor cells (MDSCs) to PD-1 on the surface of tumour-specific T cells induces apoptosis and depletion of TIL in MSD; (2) activated PD-1 prevents T cells from proliferating, by selectively inhibiting RAS/MEK/ERK and PI3K/AKT signalling pathways, blocking cell cycle-related gene transcription and protein expression; (3) the expression of PD-L1 on the surface of APCs promotes the transformation of CD4+ T cells into induced Tregs (iTregs) and maintains immunosuppressive function by down-regulating the levels of mTOR, AKT, S6 and ERK2 phosphorylation and up-regulating the expression of PTEN in CD4+ T cells. This is the reason why blocking the PD-1/PD-L1 signalling pathway is expected to restore the function of the effector CD8+ T cells, while suppressing the function of the Tregs and MDSCs, improving the anti-tumour effect of the immune system [46].

This type of immunotherapy has been approved by the FDA for the treatment of melanoma, non-small cell lung cancer, colon and rectal cancer, Hodgkin’s lymphoma, Merkel cell carcinoma, head and neck cancer, and bladder cancer [77].

• Combination of immune checkpoint inhibitors (ICIs):

The synergistic combination of monoclonal anti-CTLA-4 and anti-PD-1 antibodies is also used for the treatment of advanced melanoma, metastatic colorectal cancer that is deficient in highly unstable microsatellite repair, and colon and rectal cancer, as it has been shown to improve the overall patient response rate. For this reason, cytokines are being included in combined clinical trials with monoclonal anti-PD-1 and anti-PD-L1 antibodies [77].

On the other hand, the use of immune checkpoint inhibitors presents some problems, such as the appearance of primary and adaptive resistances to ICI monotherapy in some patients. It is therefore important to combine ICIs with other types of anti-tumoral treatment such as chemotherapy or radiotherapy, thus increasing their effectiveness. Another limitation is that some cancers do not respond to PD-1/PD-L1 immunotherapy and systemic administration of these inhibitors has immune-related adverse effects (irAE) [46].

Since the approval of ipilimumab, a CTLA-4-blocking antibody, by the FDA in 2011 for the treatment of metastatic melanoma [164], six other checkpoint inhibitory antibodies, in this case targeting the PD-1/PD-L1 axis, have been approved: nivolumab, pembrolizumab, cemiplimab, atezolizumab, durvalumab, and avelumab. These ICIs act on a wide range of cancers: melanoma, NSCLC, hepatocellular carcinoma, squamous cell head and neck carcinoma, Hodgkin’s lymphoma, urothelial carcinoma, etc. [165,166,167,168]. In 2015, the FDA approved the combination of ipilimumab with nivolumab (anti-PD-1 antibody), as it showed an improved response rate compared to any monotherapy in the treatment of melanoma [169]. In addition, there are several active clinical trials of ICI combination therapies [170,171]. The identification and validation of more reliable biomarkers would allow for more appropriate selection of patients with cancer that would improve the response rate [172].

### 3.4. Limitations of Immunotherapy

**Figure 11:**
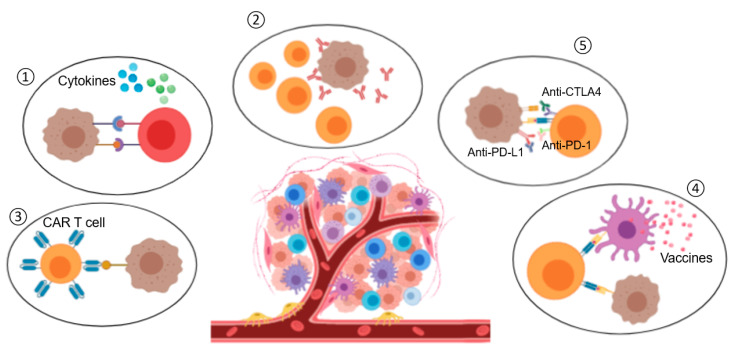
Summary of the different immunotherapies described above. (1) Cytokines, (2) monoclonal antibodies (mAb), (3) CAR-T cells, (4) onco-vaccines and (5) ICIs [173].

The previously described immunotherapy strategies (Figure 11) have some limitations and face different challenges.

Although cytokines were the first approach for immunotherapy introduced in the clinic, they also have some drawbacks. Cytokine treatments consist of high-dose injections, as their half-life is short, resulting in vascular leakage and cytokine release syndrome. In addition, cytokines can promote the survival of regulatory T cells and induce death in stimulated T cells, resulting in autoimmunity against healthy tissues [174].

As for agonist antibodies, they have dose-limiting toxicities, as do cytokines, since they can induce activity on unwanted immune cell subtypes, and immune activity towards healthy cells. In addition, some of these antibodies induce regulatory activity on T cells [175]. Therefore, it is necessary to evaluate dose-associated toxicities and develop delivery platforms. One example is anti-4-1BB antibodies, which—when anchored to liposomal NPs—have a higher intra-tumoral accumulation and lower toxicity than antibodies released freely in mouse models [176].

In the case of CAR-T cells, unlike other treatments, they are unique therapies and the cells can maintain their activity for several years after injection. Despite this, the long-term effects of therapy with CAR-T cells are still being investigated [177]. Other disadvantages of this therapy are that the production of CAR-T cells is expensive, technically complex and time-consuming. In certain tumours, especially solid tumours, depending on their microenvironment, the infused cells do not persist and need combination therapies and new drug delivery systems to improve T-cell survival [175].

CAR-T cells and TCR cells can cause cytokine release syndrome and neurotoxicity [177]. Another problem is making these modified cells effective in solid tumours. One of the solid tumours that has been successfully treated with CAR-T cells is glioblastoma [58], but it expresses the target antigen (EGFRvIII) at much higher levels in the tumour cells than normal cells, which is unusual. As for T cells with high affinity TCR, their toxicity is difficult to predict [178].

In the case of vaccines, those based on DCs have demonstrated high safety profiles, while in clinical trials they have shown a lack of efficacy [179]. The efficacy could be improved by identifying subsets of dendritic cells expressing high levels of specific antigens and by improving the supply to the lymph nodes [180]. As for DNA- or RNA-based vaccines, the former have been tested in clinical trials but are often not successful due to nuclear supply barriers and immunogenicity [181]. mRNA vaccines also have some drawbacks, such as the fact that mRNA can be degraded by nucleases and not internalized into cells. The use of delivery pathways to mediate intracellular internalization may be a good option [182]. Neoantigen vaccines cover an unlimited number of neoantigens, but delivery platforms can improve their efficacy by increasing the stability of the encapsulated molecules and by housing several neoantigens within one platform to treat heterogeneous cancers [175].

For ICIs administered by the systemic route, they can have serious side effects in several organs [183,184]. In addition, many patients do not respond to this treatment, which may be due to a low number of tumour-infiltrating T cells, dysregulation of the checkpoint axes or adapted resistance to checkpoint inhibition [185]. Different tumour microenvironments also have different mechanisms of immunosuppression that require new approaches for effective treatment.

The TME, in the case of solid tumours, is a challenge in the implementation of the above-mentioned immunotherapies. The TME of these tumours can be classified as immunologically “hot” (high immunogenicity) or “cold” (low immunogenicity), with high or low levels of cytotoxic lymphocyte infiltration, respectively. “Hot” tumours have better responses to ICIs than “cold” tumours; then, delivery technologies might be exploited to modulate immunogenicity for “cold” tumours [186].

Another drawback of immunotherapies is related to the systemic toxicity, which can be reduced by delivery platforms by limiting drug exposure in specific tissues, thus allowing for the delivery of otherwise highly toxic combination therapies [187]. The study by Wantong Song et al. shows that NPs allow for the administration of combination immunotherapy treatments, making “cold” tumours susceptible to immunotherapy [188]. Nanomedicines can be designed to respond to the tumour microenvironment and increase site penetration in both “hot” and “cold” solid tumours, overcoming the limitations of immunotherapy [189].

Immunotherapies that require intracellular administration, such as genetic vaccines, must overcome extra- and intracellular barriers with minimal systemic toxicity [190]. Administration and delivery technologies, such as NPs, would allow for the therapeutic burden of such immunotherapies to be encapsulated and protected until they can be released into the cytosol of the target cells [191,192].

## 4. Nanomedicine and Immunotherapy: Synergy Combination

**Figure 12:**
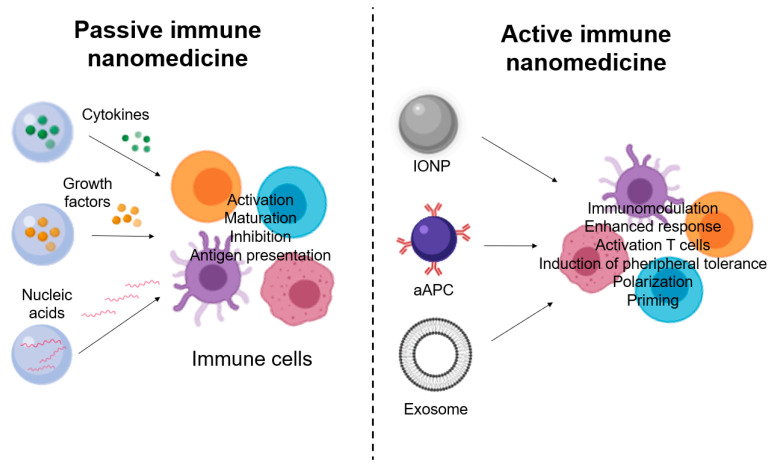
Classification of nanomedicines according to the function caused/promoted on the immune cells [193].

In order to improve the effectiveness and minimize the toxicity associated with cancer immunotherapy, new strategies have been attempted, including the use of nanomaterials to increase host immunity. Nanomedicine can play a role in improving both active and passive immunotherapy, depending on the functions for which the different NPs have been designed and the processes in which they participate (Figure 12). These NPs can be designed as delivery platforms for immunotherapy, i.e., as delivery vehicles which allow for more efficient and specific transport of immunostimulatory agents, which we will call passive nanomedicine; or they can be designed with nanomaterials which have intrinsic immunomodulatory properties that help to increase anti-tumour immune responses by selectively regulating signalling pathways in different immune cell populations, called active nanomedicine.

### 4.1. Passive Immune Nanomedicine

Passive immune nanomedicine involves NPs conjugated with growth factors, cytokines and nucleic acids, which intervene by stimulating the maturation, activation or inhibition of some cells of the innate immune response, as well as enhancing the antigenic presentation, with the final objective of activating the adaptive immune response.

Synthetic and natural NPs have physical and chemical ideal properties that make them optimal drug carrier platforms for targeted delivery, such as allowing their pharmacokinetic and pharmacodynamic properties to be modified without altering their anti-tumour effect. The surface of these NPs is directly modified with chemical motifs for selective and/or oriented coupling/immobilization of different biomolecular targets. Commonly, among chemical moieties, a plethora of biomolecules could be bound to the surfaces such as: antibodies, peptides or recombinant proteins, DNA probes, in order to facilitate the selective accumulation of drugs within the tumour tissues when they are released from the internal nucleus of the NP, where they are encapsulated [193].

Here, a few of the most representative NPs involved in the multiple functions mentioned above will be further discussed in order to reflect the advantages in oncotherapy.

Lipid-based and polymer-based NPs allow effective delivery of antigens or viral peptides to APC to stimulate memory T-cell responses to tumours. Self-assembled NPs increase the production of inflammatory cytokines such as IL-2 and IFN-γ in activated leukocytes, generating powerful immune responses to low immunogenic tumours.

In this area, another described application of NPs is to produce direct delivery of cytokines, cell growth factors or stimulant cocktails to activate specific or particular functions of immune cells. NPs capable of delivering nucleic acids such as siRNAs or Cas9 mRNAs have also begun to be used to intervene in transcriptional modifications or repair genes associated with disease [193].

One example is the reprogramming of circulating T cells to the anti-tumour phenotype by inserting chimeric antigen receptor (CAR) genes for leukemia into the nucleus using containing synthetic DNA coupled to NPs [194], which offers some advantages over current CAR-T cell therapy, such as replacing the ex vivo expansion of T cells isolated from the patient. In the case of RNA, sequences encoding viral neo-antigens or mutants are used [193], and it is encapsulated in lipid NPs that protect it from degradation by extracellular ribonuclease, ensuring its internalization into APCs so that they express in vivo engineered antigenic peptides [195]. The use of the latter type of NPs has been shown to induce anti-tumour effects and memory T cells, through the activation of INF-α, and induced strong anti-tumour anti-specific responses in three melanoma patients [51].

NPs present another potential advantage, namely targeted immunization, since they are mostly captured (by different mechanisms such as phagocytosis, pinocytosis, and endocytosis) by innate immune cells such as macrophages, monocytes and dendritic cells. The surface of these NPs is a binding substrate for serum proteins such as albumin, apolipoproteins and complement system, forming a biological corona that interacts with different receptors that are expressed in membrane of professional phagocytic cells (i.e., macrophages, DCs, etc.). Although this non-specific absorption by phagocytic cells may be a disadvantage in the case of conventional nanomedicine because it reduces the accessibility and availability of encapsulated drugs in tumour tissues; however in the case of immune nanomedicine it may be an advantage, since these NPs can thus reach lymphoid organs such as the spleen and produce their immunomodulatory effect there.

These properties make NPs outstanding candidates for the administration of tumour vaccines and/or vaccine adjuvants, as they improve their potential while reducing side effects by preventing the systemic distribution of these adjuvants and prolonging their role in lymph node drainage [193].

The intervention of NPs in the different processes of innate immunity can improve the efficacy of passive immunotherapy, and it is therefore necessary to develop new strategies to exploit the full potential of nanomaterials combined with different immunotherapeutics. Passive nanomedicine is an approach that offers many possibilities for improving cancer treatment, which should be further investigated with the aim of transferring its benefits to the clinic.

### 4.2. Active Immune Nanomedicine

In active immune nanomedicine, different synthetic nanoconstructions or natural nanostructures are used which, due to their intrinsic immunomodulating properties, increase the responses of immune cells, in this case interacting with adaptive immunity cells in a more specific way. In this case, there are many different designs and modifications of NPs that can be used. Some are described below as conjugated NPs, exosomes, artificial antigen-presenting cells or iron oxide NPs (IONPs), among the most promising strategies.

#### 4.2.1. NP Conjugates

One of the novel applications of NPs is their possible role as immunomodulating agents for the treatment of patients with cancer or auto-immune disorders. Liposomal or polymer NPs are designed to mimic biological interactions between APCs and T cells, which can also act as specific subcellular granules to promote anti-tumour immunity [193]. One example is polydimethylsiloxane (PDMS) particles, modified with antibodies to CD3 and CD28, which activate and enhance the in vitro expansion of TCD4+ and CD8+ cells [196].

For instance, NPs could also be designed for direct dependency on immune cells to target and attack different tumour cells [193]. For example, different NPs loaded with chemotherapeutic agents that reduce local recurrences may be delivered via neutrophils, as these cells will be recruited to the tumour resection bed by the inflammatory cytokines released after surgery in the case of brain tumours [197]. Other types of innate immune response cells, such as platelets conjugated with anti-PD-L1 antibodies on their membrane, which also accumulate in the tumour bed after surgery, can also be used to reduce local recurrence [198].

It should be noted that various studies have revealed the importance of the size, shape, density, rigidity and spatial organization of the MHC, among other characteristics [193], since it has been shown that, in the case of NPs used as a substrate for artificial APCs, their size is fundamental for the activation of T cells [2,199].

Another employed NPs are super-paramagnetic, such as those based on fucoidan-dextran, which can be modified with antibodies that inhibit PD-L1 and activate T cells to generate a multifunctional complex. Hence, magnetic field orientation in vivo towards the tumour is achieved by the properties of the nucleus and its effect outside the nucleus is minimised, while the tumour immune response is enhanced by the above-mentioned antibodies [200].

Beyond the modification of nanomaterial compositions, the NPs can also be engineered to enhance tumour cell phagocytosis and subsequent antigenic presentation by macrophages. For example, in HER2-positive breast cancer, bio-specific nanoparticle systems that recruit macrophages to tumour cells with the HER2 receptor can be used [201].

Taking into account the multiple possibilities of conjugation of the nanomaterials and biomolecules described above to design NPs, some of the most important ones, such as aAPCs or iron oxide NPs, are described below. In addition, exosomes, which are nanovesicules that come from cells and transmit information between tissue microenvironments, will be reviewed [202]. In other words, exosomes are vesicles of completely natural origin that can also be used in nanomedicine.

#### 4.2.2. Exosomes

As it was described previously, exosomes are also considered NPs that are originate from cells and transmit information between tissue microenvironments and can influence the function and differentiation of target cells. They are secreted by all cell types, including immune cells (such as B and T cells, DC cells), cancer cells, stem cells and endothelial cells; in addition, exosomes and are present in the human proximal fluids such as blood, urine and breast milk. In general, exosomes are constitutively released by tumour cells or in a regulated manner by immune cells (i.e., B cells). Exosomes biogenesis is produced by internal germination of late endosomes and produce multivessel bodies that fuse with the plasma membrane and are released into the microenvironment [203]. Structurally exosomes are composed of a lipid bilayer expressing ligands and surface receptors, which contains a hydrophilic nucleus. In the nucleus, there is a high rich content from RNA, proteins and other components that come from the source cells. Thus, exosomes carry information in the form of mRNA and miRNA that will correspond to the normal or pathogenic processes of the cells from which they come [202], such as the elimination of unwanted proteins, the presentation of antigens, genetic exchange, immune responses, angiogenesis, inflammation, tumour metastasis and the spread of pathogens or oncogenes [204,205].

Regarding the content on membrane proteins, exosomes contain very interested ligands such as integrines, tetraspanines, and receptors in native conformations, including the co-receptors needed for in vivo signalling [206], among others. The adhesion molecules (i.e., integrines, selectins, etc.) contained in exosomes are known to be expressed on the cells from which they originate; for example, DC-derived exosomes express CD80 and CD86 [207], B-cell derivatives express CD19 [208]. From the proteome point of view, exosome proteomes have been analysed in several studies because, although they constitute only a small part of the total plasma proteome, they are enriched in altered proteins under different pathological conditions and might therefore be considered diagnostic markers [203,209]. Therefore, in lung cancer, colorectal cancer and diabetes, specific expression patterns of serum miRNA have been identified as biomarkers for the detection of these diseases in human physiological proximal fluids [210].

Bearing in mind all these inherent properties of exosomes, they seem ideal biological nano-carriers. Moreover, due to origin, exosomes present biocompatible, such as immune tolerance, which allows them to avoid elimination through adaptive response [206]. They also escape phagocytosis, because they could fuse with cell membranes and avoid lysosome envelopes, and are more stable in the blood [211]. These exosomes can be modified either endogenously at the cellular level or exogenously in cell cultures. Endogenous modification is based on modifying exosome components, such as proteins, at the level of production of the cell from which they originate [206]. Exogenous modifications are important in understanding the extent to which the contents and function of exosomes of different biological origins could be manipulated. These exogenous modifications provide information on how exosomes target and interact with tissue-specific microenvironments in vivo, which would allow for new applications in diagnosis and therapy. The structure of exosomes allows for three types of exogenous modifications: 1.-modifying exosome surface molecules to allow specific targeting and monitoring of exosomes, 2.-loading hydrophobic therapies onto the membrane, and 3.-loading hydrophilic drugs or therapeutic cargo into the nucleus. These modifications facilitate the use of exosomes as nanomedicine approaches in immune-onco-therapy [202].

One of the described applications, it is based on the pre-existing surface receptors themselves, which could be also adapted for use in therapeutic applications. In one recent study, it was shown that mesenchymal stem cell (MSC) exosomes can transmit membrane and ligand receptors to attenuate the function of self-reactive CD4 T cells isolated from mice with experimental autoimmune encephalitis. The ligand PD-L1, TGF-β and galectin-1 were transferred to the T cells and decreased secretion of IL-17 and IFN-γ by 50% by the T cells after treatment with the exosomes [212].

MSCs produce a greater number of exosomes than other cell types and this production is not compromised in terms of quantity or quality thanks to the immortalization of these cells to generate permanent cell lines that guarantee the reproducible and sustainable production of exosomes from MSCs [213]. These exosomes, in addition to surface markers CD9 and CD81, express adhesion molecules that are also expressed on the MSC membrane, such as CD29, CD44 and CD73. MSCs recruit and regulate T cells, either by cell to cell contact or paracrine. Cytokine secretion and ligand–receptor inhibitory interactions are believed to be an important function of MSCs [203]. Exosomes derived from these cells act as mediators that induce peripheral tolerance of self-reactive cells by carrying MSC-specific tolerance molecules such as PD-L1, Gal-1 and TGF- β. These exosomes have been shown to inhibit the proliferation of self-reactive lymphocytes and promote the secretion of anti-inflammatory cytokines such as IL-10 and TGF-β, among others [214]. Therefore, MSC-derived exosomes are mediators that induce peripheral tolerance and modulate immune responses, and could therefore be used in the treatment of auto-immune diseases [81]. These exosomes have also been used in graft-versus-host disease (GVDH), which has been shown to delay its appearance in mouse models and to increase Tregs cells [215].

Another relevant study, described by Bo Yu et al., was based on the idea that MSCs have different effects on tumour growth, as they may favor tumour initiation or inhibit the progression of established tumours. Thus, exosomes released by MSCs also have varied effects [203]. One effect is the increased incidence and growth of tumours induced by certain cell lines, which indicates that MSC-derived exosomes promote tumour progression as do MSCs in vivo [216]. Another study showed that MSC-derived exosomes suppress tumour progression and angiogenesis by negatively regulating VEGF expression in in vitro and in vivo tumours. The miRNA-16 is believed to be responsible for the anti-angiogenic effect, as MSC-derived exosomes are enriched in this miRNA which targets VEGF [217].

Another exosome modification strategy is using in RNA administration mediated by in exosome. Exosomes have the inherent ability to transmit mRNA and miRNA between cells [218]. Among other methods, electroporation facilitates the loading of exogenous siRNA into exosomes. The efficiency of electroporation depends on the concentration of exosomes and the applied voltage. Using this method, Wahlgren et al. found that plasma-derived exosomes loaded with MAPK-1 siRNAs suppressed the levels of MAPK-1 mRNAs in monocytes and lymphocytes [219].

Momen-Heravi et al. charged B-cell exosomes with the miRNA-155 inhibitor by electroporation. When cells were stimulated with LPS, miRNA-155 increased their production of TNF-α. Exosomes loaded with the miRNA-155 inhibitor were able to reduce the production of TNF-α by LPS-treated macrophages. This strategy allows reducing the negative inflammatory component in different disease processes. The importance of choosing the correct exosome subpopulations for the therapeutic application of interest is highlighted. In this case, the isolation of exosomes was performed using anti-CD36 immunomagnetic microspheres [220]. This type of isolation and enrichment is very useful to separate exosome subpopulations for biomarker studies [221].

Exosomes may also be used as immunotolerant nano-carriers for hydrophilic chemotherapeutic load, such as doxorubicin [202]. Tian et al. designed immunotolerant immature dendritic cell (iDC) exosomes that expressed a chimeric Lamp2b fusion protein and the integrin-specific iRGD peptide α-V. Electroporation was used to load doxorubicin into the exosomes and the encapsulation efficiency was 20%. The iDC exosomes were able to target and accumulate in breast tumours expressing α-V integrin in mice and inhibit their growth. In contrast, free doxorubicin or untargeted doxorubicin exosomes had no effect on tumour growth. In addition, tumour growth inhibition with iDC exosomes did not result in observable toxicity and therefore the use of iDC exosomes as biocompatible nanoporters was validated [222].

Another recent study, it has also been conducted with modified exosomes to treat NSCLC. Here, exosomes loaded with paclitaxel were modified with PEG and AA (ligand) to increase blood circulation time and attack lung metastases. In this way, the drug selectively targets the target cancer cells and increases the survival rate of patients with lung cancer [223].

B-lymphocyte-derived exosomes have also been shown to have immunomodulatory function, triggering specific CD4+ T-cell responses and thus performing a role as transporters of MHC class II peptide complexes between immune cells [224]. In the case of DC-derived exosomes, they have been shown to express MHC class I, class II and co-stimulatory T-cell molecules and to suppress the growth of T-cell-dependent murine tumours [225]. For this reason, these exosomes may begin to be considered as cell-free “vaccines” in cancer immunotherapy [226].

Attempts are still being made to determine the endogenous function of the various exosome subtypes and subpopulations. This is even more important in the design of nano-carriers with tumour exosome subtypes that may have a pathogenic burden, which must be neutralized so that it does not impede the therapeutic efficacy of these exosomes [206]. In contrast, tumour antigen retention may also be beneficial for the development of tumour exosome-based immunotherapies [227]. The various therapeutic applications will require the selection of the optimal exosome subtype for conversion to nano-carriers and this requires an understanding of the normal function of exosomes and the ability to predict the function of modified exosomes.

#### 4.2.3. Artificial Antigen Presentation Cells (aAPC)

Artificial antigen-presenting cells (aAPC) deliver stimulation signals to cytotoxic T cells and are a powerful tool for active and adoptive immunotherapy [228].

In fact, the induction of specific cytotoxic T cell (CTL) responses is a potent therapy against pathogens and tumours. Specific CTLs produce robust responses and generate long-term memory [229]. As an active immunotherapy, CTLs can be activated in vivo.

Two signals are required from APCs for T cell activation, the first being a related antigenic peptide presented on MHC molecules that binds to the TCR, and the second a series of co-stimulatory receptors that modulate T cell response [230]. In immunotherapy, modified NPs that function as artificial antigen-presenting cells (aAPCs) are being used to rapidly expand tumour-specific T cells from naïve precursors and responses to predicted neo-epitopes [231].

Naïve tumour-specific precursors are rare and APC-based methods for the expansion of naïve tumour-specific cells will require continued stimulation during multiple tabletop sessions, followed by T-cell selection and subcloning to generate the number of tumour-specific cells required for adoptive immunotherapy. Therefore, the development of the ideal T-cell expansion platform is required, which generates robust expansion, minimizing culture time and costs [231].

As an example, the study described by Karlo Perica et al., iron-dextran NPs were used and a chimeric immunoglobulin-MHC dimer (MHC-Ig) loaded with a specific peptide is used to generate signal 1 and B7.1 (natural T-cell receptor ligand CD28) or an activating antibody against CD28 is used for signal 2. These molecules are chemically bonded to the surface of the microspheres to generate these aAPCs. It was found that the aAPCs induced antigen-specific T cell expansion in vitro and that both signals were essential for optimal expansion, and also induced anti-tumour activity in vivo. In addition, the amount and density of antigen presented by APCs are known to influence T cell behaviour, proliferation and cell death, and therefore are important parameters to consider in aAPC stimulation [228].

The potential sites where aAPCs may be most effective are the lymph nodes, where the naive and memory T cells are found, and the site of the tumour [228]. In addition, aAPCs may overcome one of the major obstacles in cancer immunotherapy: the tumour’s immunosuppressive microenvironment. This is because they deliver the immunostimulatory signal in situ [232]. aAPCs are known to activate T cells by specific receptor-ligand bonds at the cell-sphere interface, but such interactions are not defined at the nanoscale level [233].

Although nanoscale aAPCs have been shown to induce anti-tumour naïve T-cell populations in vivo, the ability of these nanostructures to mediate the rejection of established tumours in highly immunosuppressive microenvironments has not been determined. It is not yet well-known whether a local stimulating signal could overcome several stages of tumour immunosuppression, or even whether aAPC-based stimulation could be enhanced by other immunomodulatory therapies such as checkpoint blocking strategies [228].

Although autologous APCs (DC, monocytes and activated B cells) have originally been used to generate tumour-specific T cells in vitro, this requires regular access to patients’ blood and, in addition, the quantity and quality of each patient’s autologous APCs is variable. APCAs overcome these problems, are easy to produce and allow reliable expansion of antigen-specific T-cell populations, as we have already seen, making them a promising technology for cancer immunotherapy [234,235].

#### 4.2.4. Iron Oxide NPs (IOPNs)

Currently, there is a growing requirement for image-guided cancer therapy to design personalised therapies in cancer patients, for which advances in the translational development of IONPs may have a significant impact on the clinical and prognostic outcome of these cancer patients [236]. Several approaches based on IONPs have super-paramagnetic properties that are very useful in MRI and are used as contrast agents for diagnostic applications [237].

In recent years, several studies have been conducted to improve the properties of magnetic IONPs with the aim of making them suitable for human biomedical applications, in particular for immunotherapies. One example is surface modifications to reduce non-specific absorption of IONPs by macrophages in the reticuloendothelial system, such as polyethylene glycol (PEG) coating. To increase the efficiency of delivery, magnetic IONPs have been conjugated with different targeting ligands (antibodies, peptides, natural ligands, small molecules, etc.) directed to highly expressed cell receptors on tumour vasculatures, stromal cells and tumour cells [236]. Many preclinical studies have been performed with ligand conjugated IONPs targeting tumours with therapeutic agents for disease detection and treatment applications [238], and the effects on tumour imaging of these types of IONPs have been demonstrated in mouse models [239]. Appropriate targeting ligands and surface modifications have been shown to result in improved accumulation of IONPs in tumour tissues in animal models, while reducing nonspecific accumulation in the liver and spleen [240]. In contrast, IONPs that have been approved by the FDA are untargeted IONPs, and they have been used in humans as contrast agents in MRIs [236].

The properties of IONPs provide an enhanced effect of MRI contrast so that through this methodology drug delivery can be controlled, treatment responses evaluated, and drug delivery controlled by the external magnetic field [241]. Therefore, IONPs are a good candidate for the development of new tumour imaging, targeted drug delivery, and image-guided therapy, which have great potential in new clinical applications.

### 4.3. NP Biosafety: A Critical Aspect in Nanomedicine and Immunotherapy

To guarantee the effective and safe use of nanomaterials used in Nanomedicine, it is necessary to characterise the interaction between a material and the biological system involved. These are biocompatibility studies that have to be performed with a focus on the environment in which the biomaterial will be administered [242]. To ensure the drug delivery, it is necessary to evaluate this biocompatibility, ensuring the safe release of the medicine and minimizing its toxicity. For example, NPs that have not been modified on their surface are absorbed by phagocytic cells, which can lead to undesirable interactions with the immune system, decreasing the bioavailability of the drug [243].

Biocompatibility is defined as “the ability of a material to function with an appropriate host response in a specific situation” [244]. The employed material has to fulfil its intended functions, the reaction induced has to be appropriate to the intended application, and the nature of the reaction to a material and its suitability may be different in different contexts. The high degree of compatibility is achieved when a material interacts with the body without causing toxic, immunogenic or carcinogenic responses [243]. Importantly, biocompatibility is anatomically dependent, so a biomaterial can cause adverse effects in one type of tissue and will not cause the same response in another [245,246]. The half-life of exposure is also determinant and, therefore, the clearance of each NPs as well. Biocompatibility is subjective, as it is based on the risk-benefit ratio.

Currently, a few studies on biological processes in response to foreign materials or on the nature of the methods available for biocompatibility, so there is a strong need to evaluate the biocompatibility of each material individually and specifically for each tissue and application [243]. An example of this type of studies is the analysis realized by Mikhail V. Zyuzin et al., who proved the immunocompatibility of polyelectrolyte capsules synthesized by layer-by-layer deposition through the incubation of different cell lines with this capsules [247].

NPs are delivered into the bloodstream and are therefore exposed to many biomolecules that will give rise to a protein crown around them [248]. This causes NPs to undergo changes in their physicochemical properties, and therefore it is necessary to study NP–protein interactions in nanomedicine. Proteins in the biological environment are adsorbed to NPs by affinity and protein–protein interactions [2]. The first proteins to bind are those found in high concentrations, although they have low affinity, and are subsequently replaced by proteins with higher affinity that are found in lower concentrations. This phenomenon is called the Vroman effect [249]. The protein crown is classified into hard or soft crown depending on the duration of protein replacement. The hard crown is formed by high affinity proteins with a long exchange time and is the innermost layer. The soft crown is made up of proteins with low affinity and a rapid exchange of proteins [250].

Both the characteristics of NPs and protein concentration, and the biological environment will determine the formation of the protein crown. Therefore, it is important to understand the relationship between the properties of nanomaterials and the biological environment to understand the behaviour and viability of the NPs used.

### 4.4. Immunogenic Cell Death: A Merge Point of Nanomedicine and Immunotherapy

The maintenance of homeostasis in the human body involves the continuous replacement of different cell compartments, which does not activate the immune system under normal conditions. Instead, the death of some pathogen-infected cells may generate a strong immune response, further establishing a long-term immune memory. Conventionally, only the “self/non-self” model was used to differentiate homeostatic from pathogen-associated cell death, respectively. However, in the 1990s it was demonstrated that some endogenous entities were capable of initiating an immune response in certain circumstances. This means that there would be another factor other than antigenicity that would determine the immunogenic capacity of the different forms of cell death [251].

The Microbe-Associated Molecular Patterns, called MAMPs, are detected by multiple cells of the innate immune system, such as monocytes, macrophages and DC, before the pathogens activate the adaptive response [252]. MAMPs function as adjuvants, interacting with PRRs, which allow the establishment of the first line of defense while generating favourable conditions to initiate the specific immune response [253]. Signalling through PRRs is of great importance, since these receptors are activated by Damage-Associated Molecular Patterns (DAMPs) (Table 1). DAMPs are produced by cells that are in the process of dying and act as adjuvants, informing the body of the danger situation [254]. Under normal conditions, these DAMPs do not activate the adaptive immune response, but when the dying cells are highly antigenic, this occurs, as new antigenic epitopes (“called neo-epitopes) are detected that have not previously produced tolerance. These neo-epitopes can be expressed from microbial genes or from mutated host genes, as in the case of oncogenesis [255]. Therefore, the other factor determining cell death immunogenicity is adjuvancy, which involves MAMPs and DAMPs [251]. Thus, tumours with a high mutational load respond better to some types of immunotherapy, such as immune checkpoint inhibitors (ICIs), than tumours with a low number of somatic mutations [156].

**Figure 13:**
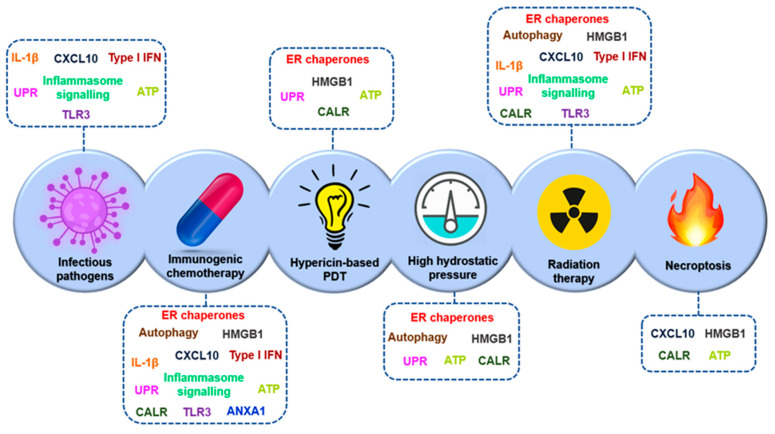
Schematic representation of Immunogenic Cell Death (ICD) classification and their associated DAMPs.

In the past, cell death was classified only in apoptosis as a physiological process and in necrosis as a pathological and immunogenic process. Nowadays, it is now known that these differences between the two processes are not as clear, as regulated forms of necrosis are involved in tissue development and homeostasis, and apoptotic cells can trigger antigen-specific immune responses [251]. ICD is therefore a type of immune-stimulatory apoptosis that is characterised by the ability of dying cells to elicit powerful adaptive immune responses to altered auto-antigens/neo-epitope derived from tumour cells in the case of cancer [256]. Based on a specific panel of multiple DAMPs four types of ICDs have been described (Figure 13):

1. Pathogen-induced ICD: It is defined as a defence mechanism against pathogens, such as obligatory intracellular bacteria and viruses. After infection, the cells detect MAMPs through specific PRRs, which will send danger signals to neighbouring cells. Intracellular hazard signalling is generated, which will activate autophagy, and microenvironmental hazard signalling, which will induce the secretion of pro-inflammatory cytokines, including TNF and type I interferons. The adaptive immune response is activated when infected cells die and their bodies are internalized in APC, which will present the various non-self-antigenic epitopes in MHC molecules, thus activating CD8+ and CD4+ T cells [251].
2. ICD caused by chemotherapy and/or targeted onco-therapy: Exposure to certain chemotherapeutic agents used in the clinic has been shown to produce ICD in mouse tumour cells [257]. This ICD is based on eIF2A phosphorylation-dependent exposure of endoplasmic reticulum chaperones in the membrane of these tumour cells. Processes such as autophagy-mediated ATP secretion, IFN type I activation, secretion of chemokine ligands such as CXCL10, etc. are also involved. These processes also occur in human tumour cells after chemotherapy. Chemotherapeutic agents that are unable to promote the release of DAMPs do not produce ICD. The degree of antigenicity among tumour cells is very heterogeneous, which may condition the activation of adaptive immunity after ICD. However, the lower level of mutational load associated with oncogenesis has been found to be sufficient to activate this immunogenicity, which is due to the fact that tumour cells express neoantigens that are different from their own and are therefore not subject to tolerance [251,258].
3. ICD activated by physical signals: There are three physical interventions that trigger ICDs: irradiation, hypericin-based photodynamic therapy (PDT), and high hydrostatic pressure [251,258]. DCs loaded with irradiated tumour cells have been shown to produce strong immune responses in mice and cancer patients [259]. ICD due to hypericin-based PDT or high hydrostatic pressure shows exposure of ER chaperones on the plasma membrane, ATP secretion, and high-mobility group box 1 (HMGB1) [260,261]. DCs exposed to cells suffering from these two types of ICDs induce positive regulation of DC-activation markers and pro-inflammatory cytokine secretion, resulting in priming of tumour-specific CD8+ T cells [251].
4. Necroptotic ICD: This is a form of programmed cell death, initiated by phosphorylation catalyzed by the serine/threonine kinase 3 (RIPK3) protein, which activates the pseudokinase mixed lineage kinase domain-like (MLKL) receptor, which forms oligomers that produce irreversible plasma membrane permeation [262]. Necroptosis is highly pro-inflammatory and is also capable of activating the adaptive immune system, generating a specific antigen response [251]. This has been demonstrated in studies with the mouse cell lines TC-1 and EL4 of lung carcinoma and CT26 of colorectal carcinoma, which were exposed to necroptosis inducers [263,264].

The characteristics of the ICD, such as the exposure of CRT and other ER proteins on the cell surface, the release of HMGB1 or the secretion of ATP, allow for the prediction of the capacity of anti-cancer drugs to stimulate therapeutic immune responses by ICD [265].

For example, calreticulin (CRT) is an ER-associated chaperone involved in various functions, such as MHC-I assembly or calcium homeostasis. Tumour cells that undergo chemotherapy-induced cell death expose CRT on their surface, causing CRT to internalize tumour material and present tumour antigens, activating tumour-specific cytotoxic T cells [265]. Tumours that do not properly expose CRT have been shown to have reduced efficacy of chemotherapy, so this immunogenic signal is necessary to obtain good immune responses [266]. In the clinic, CRT exposure is related to patient survival. In patients with non-Hodgkin’s lymphoma, the therapeutic benefit of a pulsed DC vaccine with primary lymphoma cells that undergo ICD is correlated with CRT exposure [267]. For patients with acute myeloid leukemia, CRT exposure by tumour cells is known to predict anti-tumour T-cell responses and improve patient survival [268]. Colorectal cancers that do not express CRT have a worse prognosis [269]. Therefore, the expression of CRT affects the immune responses to the cancer in an important way.

Another important factor is that the tumours are competent in autophagy, since these tumours, in response to chemotherapy, recruit macrophages, DC and T lymphocytes more effectively. This is because autophagy is essential for the immunogenic release of ATP by dying cells, which is a potent chemotherapeutic agent [265]. When autophagy is inhibited in cancer, the recruitment of immune effectors to the tumour bed fails, so it may be an escape mechanism from immune surveillance. Inhibiting enzymes that degrade ATP may improve antineoplastic therapies when autophagy is deactivated [270].

In the case of HMGB1, it is a potent pro-inflammatory stimulus whose release can be induced by most antineoplastic agents [271]. HMGB1 activates the release of pro-inflammatory cytokines by monocytes and macrophages. It has been observed that its neutralization with antibodies prevents cross-presentation of tumour antigens by DC in co-culture experiments, therefore its release is a critical determinant of ICD [272].

The induction of ICD in vivo also generates a TME dominated by Th1 and Th17 cytokines [273], which is expected to increase the efficacy of anti-tumour vaccines and therapies designed to reagent TILs, such as ICI.

But once again, these approaches also encounter some obstacles in the tumour cells. Pathogenic viruses and bacteria have developed different mechanisms to prevent the release or detection of DAMPs to escape the immune response. To this end, they express functional orthopaedics of some molecules and inhibitors of different processes that would be necessary for manifest pathogenicity, mainly limiting adjuvancy. The same occurs with tumour cells, which although they present a high antigenicity, control immunogenicity by acting on the adjuvancy, inhibiting the different processes related to the emission of DAMPs, which impairs the efficacy of treatments such as chemotherapy or immunotherapy [251].

Nanomedicine can also act at the level of ICDs and DAMPs (damage associated molecular patterns), with the aim of restraining the immunogenicity of tumour cells. The NPs can be used to enhance the “danger signals” that are released by these tumour cells. Adjuvant-charged NPs are used and placed in the cells suffering from ICD, thus promoting the transmission of these signals [274]. Another application is the targeted delivery of ICD inducers by other NPs, in the form of discs, which allow them to accumulate in the tumour and positively regulate the danger signals, thus enhancing the response of T cells to neo-antigens, tumour-associated antigens and whole tumour cells [275]. Some NPs also have intrinsic properties to induce ICD, such as gold NPs, which release endogenous immune-stimulatory molecules and facilitate DCs activation [276]. NPs combined with chemotherapy and PDT can also be used to induce ICD or to capture TAAs that are released after radiotherapy, with the goal of enhancing T-cell response when treated with ICIs such as anti-PD-L1 [277,278].

Therefore, the immunotherapeutic strategies described above could benefit from the concept of ICD, avoiding some of the drawbacks that occur in the clinic and enhancing an effective immune response, since the different molecules that determine ICD are involved in multiple processes of the immune cycle against cancer. Pre-clinical and clinical studies have already been conducted that could lay the foundations for the design of combined therapies that restore cellular immunogenicity [279,280].

The concept of ICD can also be used to identify biomarkers to predict therapeutic responses in cancer patients. The distinctive features of ICD in tissues need to be identified and correlated with immunological and clinical observations. It is also important to determine what changes in the immune infiltrate of tumours are caused by the ICD and how they affect therapeutic responses.

## 5. Biomarkers in Onco-Immunotherapy

As we have already described, immunotherapies are one of the most promising approaches to treat cancer patients, but despite the demonstrated success in a variety of malignant tumours, the responses only occur in a minority of patients. Furthermore, these treatments involve inflammatory toxicity and a high cost. Therefore, determining which patients would derive clinical benefit from immunotherapy is an important goal. This requires the identification and validation of prognostic biomarkers. The integration of multiple tumour and immune response parameters, such as protein expression, genomics and transcriptomics, may be necessary to accurately predict clinical benefit.

### 5.1. Critical Role of Predictive Biomarkers in Oncology

Progress in the field of immune-oncology has changed traditional treatment models which also the design in clinical trials in order to define objective responses to treatments. With the advent of checkpoint inhibitors, subsets of patients with treatment-resistant metastatic cancers have had long-lasting responses, although many patients still do not respond [281]. Objective responses among patients treated with single-agent regimens are seen in less than half of the patients treated, and combination checkpoint inhibitor therapy increases response rates but also toxicity and cost [282], highlighting the need to identify predictive biomarkers for outcome [281]. The importance of identifying these predictive biomarkers, and not just prognoses, lies in the need to optimise the selection of appropriate tumour types and patients for treatment with immunotherapy, in order to increase efficacy and to avoid unnecessary toxicities, high healthcare costs, etc. This can improve the selection of tumour types and patients that will benefit from immunotherapy, as well as the determination of which patients need a single therapeutic agent, several combined strategies or the development of alternative treatment strategies [282]. Although progress in biomarker research has been rapid, only a few biomarkers have proven to be clinically relevant, including PD-L1. These biomarkers are used to select patients for FDA-approved therapies, but other biomarkers are not yet well-established [281].

Currently, the development of biomarkers in onco-immunotherapy is limited because many of the targets are often inducible and with variability in time and location [282]. This is influenced by the TME and immunoedition. In the TME, there are interactions between various types of infiltrating immune cells (monocytes, granulocytes, DC, T and B cells, mast cells, NK, etc.), heterogeneous tumour cells and tumour-associated stromal cells (macrophages, fibroblasts, endothelial cells). In addition, there is a local variation in oxygenation, perfusion, electrolyte levels and tumour cells that become resistant in conditions of anoxia and lack of nutrients, which generates “microniches” within the tumour microenvironment itself. In addition, clones of tumour cells that are resistant to selective pressure may appear due to incomplete immunoedition and immune escape [283]. The importance of tumour-infiltrating lymphocytes within the tumour microenvironment has been established as containing prognostic value for cancer patients and predictive value for treatment with immunotherapy [282].

**Figure 14:**
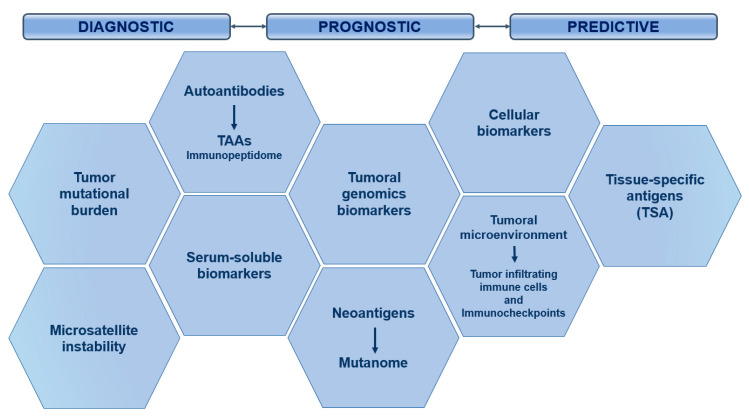
Summary of available biomarkers for Immuno-Oncology.

Therefore, to target cancer therapy, the variety of biomarkers and trials required is wide. This is due to the great diversity of immunotherapy agents with different mechanisms of action, to tumour heterogenicity, including changes in antigenic profiles over time and the location of each patient, and to the different immunosuppressive mechanisms that are activated in TEM. This complexity requires a profile of the tumour immune interface using multiparametric technologies that encompass the dimensionality and complexity of these interactions, in order to monitor and stratify cancer patients according to individual therapeutic requirements. All this complexity in turn is a rich source of biomarkers [284]. The types of potential biomarkers (Figure 14) and their possible relationship with the tumour immune cycle are described below.

### 5.2. Potential Biomarkers in Immuno-Oncology

The FDA defines the concept of biomarker as: “A characteristic that is objectively measurable and evaluable as an indicator of a normal biological process, a pathogenic process, or a pharmacological response to a therapeutic intervention” [285]. Biomarkers are all those molecules that are found in body fluids in small quantities and that are associated with specific health and/or disease processes, and are classified into three types according to their purpose: (1) diagnostic biomarkers: used to detect disease; (2) prognostic biomarkers: used to predict the course of the disease; (3) predictive biomarkers: used to predict the patient’s response to treatment. A single biomarker may meet the criteria for different uses [286].

Depending on their nature or location, there are different types of biomarkers, such as soluble factors, tumour-specific factors, host genomic factors, cellular biomarkers, or TME biomarkers [282]. The most important characteristics of the multiple types of potential biomarkers are described below.

#### 5.2.1. Serum-Soluble Biomarkers

Potential biomarkers present in serum, plasma, or peripheral blood are more useful in the clinic; because in general, they should be accurately measurable and reproducible, clinically feasible, cost-effective, and prospectively validated in randomized clinical trials [282].

Soluble serum proteins as possible biomarkers were first suggested in studies of advanced melanoma and colorectal cancer (CRC) patients with high doses of IL-2. High serum levels of IL-6 and C-reactive proteins (CRP) were identified in pre-treatment as possible prognostic markers of treatment failure and shorter overall survival in metastatic CRC after IL-2 therapy [287]. High serum levels of pre-treatment CRP predict resistance to IL-2 therapy in patients with metastatic melanoma [288]. Subsequently, in patients with advanced melanoma, pre-treatment serum VEGF and fibronectin have been shown to be inversely correlated with response to IL-2 treatment [289].

High levels of VEGF and CRP are also inversely correlated with the response of melanoma patients treated with ipilimumab. Elevated serum LDH levels are also a negative predictive value in these patients. If a decrease in LDH and CRP levels occurs during treatment with ipilimumab, at week 12 it is associated with significant disease control [290].

Another potential soluble biomarker is CD25, which has favourable results at low levels, but resistance to treatment with ipilimumab at high levels. However, it is not clear whether this CD25 is a predictive or prognostic biomarker [291].

Circulating predictive biomarkers are expected to include markers of increased type 1 immunity and cytotoxic cell activity [292]. These include cytokines such as IFNγ, IL-12, IL-2 and chemokines such as CXCR3 and CCR5 that are associated with tumour trafficking and stimulate cytotoxic functions [293]. Otherwise, the immunosuppressive pathways of MSD will be disrupted, with molecules such as IDO, MDSCs will increase and immune-regulatory pathways will be stimulated [294].

These types of biomarkers are easily measurable and can be very useful in the clinic, so their identification and validation are essential. So far, most published analyses of these types of biomarkers in immunotherapy have been retrospective [282], although important information has been obtained to determine the mechanisms of clinical benefit. Clinical trials with different approaches still need to be designed to establish the use of these biomarkers in the clinic on a routine basis.

#### 5.2.2. Cellular Biomarkers

Different types of cells in peripheral blood have been studied as prognostic and predictive factors, including T cells, NK cells, DC, macrophages and tumour cells [282]. For example, high numbers of neutrophils and monocytes in peripheral blood are associated with poor survival in metastatic melanoma and serve as prognostic factors for overall survival in IL-2 treated melanoma patients [295].

Lymphocytes are the cells that have been most studied as a predictor of response to immunotherapy [282]. Circulating tumour-reactive lymphocytes can be sampled by multiparametric immunophenotypic analysis with a focus on biomarker development. Thus, immunophenotype by multiparametric flow cytometry allows identification of biomarkers associated with persistence, establishment of antitumour memory, and improvement of clinical outcomes [296,297]. The expression of PD-1 by peripheral lymphocytes correlates with tumour load, which may serve as a biomarker for the response to immunotherapy [298]. Initial lymphopenia and rebound lymphocytosis are known to follow IL-2 treatment. There is a positive association between clinical response and the degree of lymphocytosis following immunotherapy [282].

The presence of induced autoimmunity also serves to predict the response to immunotherapy. In metastatic melanoma, spontaneous antibody formation occurs for several common tumour auto-antigens, including gp100, MAGE-3, or NY-ESO-1 [282]. Patients who are HIV-positive for NY-ESO-1 are most likely to benefit 24 weeks after treatment with ipilimumab [299].

#### 5.2.3. Specific Tumour Antibodies

In the tumour of some malignant neoplasms, B cells are found, organised in germinal centers, which results in the presence of plasma cells. Their function is not yet known, but it is assumed that they are involved in a constant immune reaction at the site of the tumour. In cancer patients, circular antigen-specific auto-antibodies (AAbs) derived from tumour can be detected, which helps to determine immunogenic targets.

Ultimately, cancer sera contain antibodies that react against autologous cell antigens, AATs. Auto-antibodies associated with a particular type of cancer target these abnormal cellular proteins that are involved in tumour transformation, so autoantibodies can be considered as reporters that identify aberrant cellular mechanisms in tumorigenesis [300]. By examining the sera of cancer patients, new TAAs can be identified, such as p62 and p90 [301,302], which have already been identified with this approach. The sensitivity and specificity of different antigen-antibody systems as markers in cancer can also be evaluated to develop TAAs array systems for diagnosis, prediction, and follow-up in cancer patients [300].

The detection of tumour-associated target-specific IgG could act as a substitute for the presence of T cells [294]. It is difficult for these auto-antibodies to have a direct antitumour role, as most of the antigens they target are intracellular [303]. In the example of checkpoint inhibitors, the presence of NY-ESO-1 specific autoantibodies are known to be associated with increased clinical benefit in patients with advanced melanoma who are treated with ipilimumab [299]. This suggests that tumour-specific antibodies may be an indicator of the presence of tumour-specific T cells in the tumour microenvironment and patients with pre-existing ability to react to tumours would be favourably disposed to immunomodulatory therapy [294].

The presence of tertiary lymphoid structures, consisting of germ-center-organised B cells, plasma cells, and T cells, is highly predictive of progression-free survival and overall survival in solid tumours such as melanoma and NSCLC [304,305]. These structures are close to the tumour tissue, so they are believed to play an important role in local immunogenicity and infiltrating B and T cells are known to have tumour specificity. When B cells isolated from NSCLC tumours differentiate in vitro to plasma cells, they produce antibodies to tumour-associated antigens such as NY-ESO-1, TP53, or XAGE-1 [305]. Therefore, these tumour antigen-specific B cells participate in immune mechanisms and are potential targets for the application of immunotherapy. Associating the presence of local antibodies with systemic humoral immunity will be key to establishing serology as a prognostic or predictive marker [294].

For patients with breast cancer who present difficult to interpret mammography, Provista Diagnostics has developed a kit called Videssa ^®^ Breast that, through a blood extraction, allows a more accurate and improved diagnosis, avoiding unnecessary biopsies. It is based on proteomic technology to analyse multiple biomarkers of serum tumour proteins and tumour-associated auto-antibodies (TAAbs) associated with cancer. This kit incorporates nine serum proteins as biomarkers and 20 TAAbs. It allows detecting the presence or absence of breast cancer in women between 25 and 75 years old, with a sensitivity of 93.3% and a specificity of 63.8%. If this test is combined with image diagnosis, 100% of breast cancers can be detected. In all trials performed so far, all breast cancers were identified at an early stage [306]. This demonstrates the importance of detecting specific AAbs for TAAs to improve the diagnosis of patients with cancer.

#### 5.2.4. Tumoral Microenvironment as Biomarker

An important biomarker within the TME is tumour infiltrating immune cells. Tumours with effector T cell infiltration have an active immune microenvironment and respond better to immunotherapy [282]. Phenotypically, two classes of TME can be distinguished: those with a high prevalence of T cells and those without T cells [307].

Inflamed T-cell tumours have large numbers of T cells in the tumour periphery, plus increased expression of T-cell activation markers, type 1 interferons, and high levels of Th1 cytokines that recruit T cells. Because the cells that promote antitumour immunity are CD8+ T cells, CD4+ Th1 cells, NK cells, and mature DCs, tumours that have this predominant infiltration pattern respond better to antitumour immunotherapy [282]. Although tumour infiltrating lymphocytes are sometimes dysfunctional, their presence indicates that there is no inhibition of recruitment [294].

Tumours with a non-inflammatory T-cell microenvironment have few or no effector T cells, but contain chronic inflammation with tumour-associated macrophages, MDSCs, CD4 + FoxP3 + regulatory T cells, and Th2 cytokines, which form an immunosuppressed microenvironment that allows tumour progression, which is associated with a poorer prognosis.

Factors that may mediate the type of phenotype presented by the TME include some soluble and tumour-derived cell-membrane factors, but the mechanisms are not yet known [282].

The prognostic importance of T cells has been seen in some solid tumours, including colorectal, hepatocellular, pancreatic, esophageal, ovarian, non-small cell lung, brain metastases, melanoma, and head and neck cancer [282,294].

Antigen-specific T-cell recognition by MHC class I tetramer staining in situ or analysis of TCR’s Vβ repertoire are used to characterise MSD T cells for their specificity [308,309]. Advances in multiplex IHC technologies in tumour tissue also provide information on the nature of immune infiltration into the tumour, depending on the type, number and qualitative characteristics of the immune cells present, and their interaction with tumour and stromal cells, which is related to disease progression and prognosis [294]. In biopsies from patients who respond favourably to checkpoint inhibition, they have a higher number of proliferating CD8+ T cells associated with high levels of expression of PD-L1 as assessed by IHC and higher expression of IFNγ as determined by the gene expression profile [310,311].

#### 5.2.5. Immunocheckpoints (ICs) as Biomarkers

Other important biomarkers being worked with are PD-1 and PD-L1. PD-1 is expressed on most tumour infiltrating T cells, including antigen-specific CD8+ T cells. PD-1 is expressed after T-cell activation and results in the elimination of T cells after they have exerted their function. PD-1 is therefore a checkpoint and serves as a marker for T-cell depletion. Its ligand, PD-L1, is expressed in melanoma tumour cells, non-small cell lung cancer, CRC, bladder cancer, gastric cancer, ovarian cancer, B-cell lymphoma, Merkel cell carcinoma and Hodgkin’s lymphoma. If the interaction between PD-1 and its ligand is inhibited by antibodies, the T cells will remain active, mediating antitumour activity [282,312].

Tumour infiltrating immune cells that express PD-1 or PD-L1 are predictive biomarkers for tumours that may respond to T-cell checkpoint blocking. Tumours that have low levels of expression of these molecules have a low response rate to this treatment [282].

Expression of PD-L1 before treatment in tumour cells and immune cells correlates with improved response rates, progression-free survival, and overall survival in pembrilizumab-treated melanoma patients [281].

But the use of PD-1 and PD-L1 still has some drawbacks, such as the expression of PD-L1 is heterogeneous and dynamic within each individual, and even its expression may be induced by activated tumour specific T cells. Its expression may also vary between the primary lesion and its metastasis. In addition, other cell types present in the tumour may express PD-L1, including lymphocytes and macrophages [282,313].

#### 5.2.6. Tumoral Genomics Biomarkers

Effective immune responses to T-cell checkpoint inhibitors in some cancers correlate with the mutational load on the tumour cell [152,314]. Many of these mutations are likely to be transient and will be influencing the process of immunoedition by exerting selective pressure on the immune system. Mutational load or the emergence of neoantigens could be predictive biomarkers for the tumour response to immunotherapy agents if this hypothesis is ultimately established [282].

In one study, the entire exome was sequenced to analyse the effect of cancer genomes on the response to ipilimumab in melanoma patients. A high mutational load and the number of non-anonymous mutations per exome were found to correlate with improved overall survival. In addition, they identified 101 motifs of tetrapeptides in nonameric peptides located in the peptide-binding cleft of MHC class I molecules. These tetrapeptides were shared exclusively by patients who had long-term clinical benefit [315]. In another study, the exome of non-small cell lung cancer treated with pembrolizumab was sequenced and a high burden of non-synonymous mutations in the tumours was seen. Clinical response correlates with the molecular signatures of tobacco-related carcinogenic mutations, increased neoantigen load, and mutations in the DNA repair pathway. Pembrolizumab improved the reactivity of neo-antigen-specific CD8+ T cells and is associated with tumour regression [152].

With expression microarray technologies, genes that play an important role in immune cell biology and are highly expressed in the tumour expression profiles of some patients have been identified. These genes reflect the relative abundance of different populations of tumour-infiltrating leukocytes. From this, robust and reproducible associations between immune gene signatures in solid tumours and clinical outcomes have been identified, providing prognostic information [294]. For example, high gene expression reflecting T, B, and NK cell involvement in metastatic melanoma is associated with prolonged overall survival and survival without metastasis [316].

Immune genes have predictive potential in the context of immunotherapy. These genes include T cell surface markers (CD3, CD277, CD27, CD38), cytotoxic factors (GZMB), and tissue-rejection-related cytokines (CXCL9, CXCL10, CCL4, CCL5) [294,317].

### 5.3. Tumour Mutational Burden as Biomarkers

In different clinical studies, a high tumour mutation burden (TMB) has been associated with better response rates and improved survival of patients treated with immunotherapies such as ICI. Therefore, TMB is beginning to be used as a biomarker of response to these immunotherapy agents [318].

TMB is the total number of somatic mutations in a defined region of a tumour genome and varies by tumour type and among patients [319,320,321].

The mutational load of a tumour contributes to its immunogenicity. Tumours that have high TMB, such as melanoma and lung cancers, are thought to be more likely to express neoantigens and induce a stronger immune response after treatment with ICI [322]. Highly mutated tumours (“hot” tumours) have a histological immune signature of depleted immunosuppressive cells and high expression of immune inhibitory molecules. Less mutated tumours (“cold” tumours) have amplified immunosuppressive cells, negative regulation of MHC molecules, and low expression of immune inhibitory molecules. The adaptive immune response is very accurate in predicting patient outcome [294], so it is important to identify whether the presence of effector T cells in MSD is related to antigen-specific T cells [152,323]. For some tumours, this parameter may be a suitable clinical biomarker for making immunotherapy treatment decisions [324,325]. STM is a quantifiable measure of the number of mutations in a tumour, which is an advantage over neoantigens, since not all mutations result in immunogenic neoantigens and it is difficult to determine which mutations may induce these neoantigens, thus new techniques and strategies are required to discover new TAAs as biomarkers [156,320].

Neo-antigens are currently more easily identified by complete exome sequencing. The sequencing of new generation tumours allows the identification of mutations and, using computer algorithms, the identification of mutated peptides that bind to MHC molecules, which helps in the choice of targets to improve the response of T cells [294].

Antigenic peptides are the result of abnormal transcription, translation of alternative open reading frames or post-translational modifications. This diversity of peptides also involves the mechanism of peptide splicing by the proteasome [326]. A variety of human leukocyte antigens are involved in the processing of antigenic peptides [156]. O-glycosylation of cancer-associated aberrant proteins can modify antigenic processing and the immune response [327], and phosphopeptides associated with MHC class I are targets of memory immunity. Phosphopeptide-specific immunity has an important role in tumour recognition and control [294,328].

TMB was first determined using complete exome sequencing, but this method is expensive and has a long response time, so panel-specific sequencing is now used. The implementation of TMB implies a solid clinical and analytical validation. In addition, bioinformatic analysis is also important for its successful implementation in the clinic, since the measurement of BTM is based on new generation sequencing (NGS) techniques [329].

For ICI therapy, expression of PD-L1 correlates with an increased response to therapy and may be a predictor [330]. In contrast, not all patients who express PD-L1 respond well to ICI treatment [331]. Therefore, other MSD factors, such as LIL, also play an important role [332].

In the study by Yu-Pei Chen et al., it was proposed to classify the different types of tumour microenvironments according to the expression of PD-L1 and the presence or absence of LILs, in order to design appropriate combination immunotherapies for cancer [322,333,334]. This study attempted to establish a classification model based on analysis of mRNA expression of PD-L1 and CD8A, and evaluated the applicability of this classification to predict response to ICI treatment, i.e., its correlation with mutation load and number of neoantigens, using RNA-seq [322].

PD-L1 positive MSDs with LILs were generally associated with high BMT or number of neoantigens in multiple tumours, so these cancers would benefit from anti-PD-1/PD-L1 therapies, as these tumours have evidence of pre-existing intra-tumoral T cells being inactivated by compromised PD-L1 [322,334]. In contrast, MSDs that have low expression of PD-L1 and little infiltration of LILs will have a worse prognosis, as no immune reaction is detected. Combination therapy to attract LILs to MSDs, along with ICI, may be a good option in these cases [335]. For MSDs with high expression of PD-L1 but low infiltration of LILs, radiotherapy- mediated immunogenic cell death to release antigens and induce T-cell responses, together with ani-PD-1/PD-L1 inhibitors, may also be a beneficial approach [322,336].

In recent years, studies evaluating TMB as a predictive marker have increased significantly for the response to ICI, demonstrating the importance of this approach in selecting patients to benefit from immunotherapy.

### 5.4. TCR diversity and MHC Molecules

Thanks to the diversity of the TCR, T cells can recognise a multitude of different epitopes through TCR–MHC interaction, which is associated with effective control of viral infections, other pathogens and tumour cells. This diversity is generated by a complex mechanism based on genetic recombination of DNA, leading to different antigenic specificities [294].

Following immunotherapy based on checkpoint inhibitors, there has been increased interest in analysing TCR diversity, as it allows for a better understanding of the patient’s immune system. TCR diversity is estimated to be 10^8^–10^15^ and can be evaluated by NS, spectrometry, qPCR multiplex or immune phenotyping. The impact and clinical outcome of immunotherapy has been shown to be related to the diversity of TCR in peripheral blood [294]. Blocking CTLA-4 with tremelimumab has been shown to diversify the peripheral T cell pool, highlighting the effect of this class of immunomodulatory antibodies [337].

The effective response generated by antigen-specific T cells after recognition of tumour antigens expressed on MHC class I molecules can be affected by germline and somatic MHC class I genotype variations [281]. Patients with advanced melanoma and NSCLC treated with checkpoint inhibitors who were heterozygous at the MHC class I locus had better overall survival than those who were homozygous [338]. This is associated with increased clonality of TCR and clonal expansion of T cells following the use of this immunotherapy. The somatic loss of MHC class I heterozygosity in these patients results in poor outcome, which is associated with impaired recognition of neo-antigens by CD8+ T cells due to structural changes at the class I locus [281].

It is necessary to analyse the diversity of TCR and MHC molecules and their interactions in order to achieve a personalised and beneficial immunotherapeutic treatment for each type of cancer, as it has been shown that the basal diversity of TCR in peripheral blood is associated with clinical outcomes [294,339].

### 5.5. Neoantigens as Biomarkers

There is clear evidence that human tumour cells express antigenic determinants (epitopes) that are recognised by patients’ autologous T cells. The short peptides that enable this recognition and the specific removal of the tumour cells occur on MHC molecules and are called immunopeptidomes. T-lymphocytes target epitopes that are formed by epigenetic, translational and post-translational alterations of tumour cells. These TAAs have been exploited for therapeutic purposes, as we have seen, with good but controversial results [340].

It was later shown that tumour cells have mutated endogenous proteins that distinguish them from other cells and can be processed into peptides, presented on the cell surface and recognised by the immune system in vivo, which recognizes them as foreign. These proteins are what are called neoantigens. Neoantigens are very specific, so targeting them would allow for immune cells to distinguish tumour cells from normal ones, thus preventing autoimmunity [340].

Results in mice and humans show that CD8+ and CD4+ T cells are reactive against neoantigens [341,342,343,344]. Central T cells are not tolerant of these neoantigens because they are tumour-specific; in fact, these neoantigen-specific T cells have a high functional avidity. In contrast, the reactivity of T cells to autoantigens is reduced and is only achieved when tolerance to these antigens is not fully developed. It has been shown that in cancer patients, TAAs are recognised by T cells with reduced functional avidity [342]. In the case of neoantigens, T cell responses will not produce autoimmune toxicity against healthy tissues, so therapeutic vaccination with neoantigens may be very promising. The problem is that neoantigens are patient-specific and it is unlikely that a vaccine targeting neoantigens shared by large groups of patients can be developed with current knowledge [345].

Neoantigen-reactive T cells have been identified in human cancers such as melanoma, leukemia, ovarian cancer, and cholangiocarcinoma [342,343,346]. For melanoma and NSCLC, TMB was correlated with clinical outcome after anti-CTLA-4 and anti-PD-1 immunotherapy, respectively [152,323]. In addition, the frequency of neoantigen-specific T cells increased in patients who responded to therapy, which correlated with a favourable clinical outcome [342,343,346]. This indicates that the recognition of neoantigens is an important factor in the response to clinical immunotherapies.

Mass sequencing can identify the spectrum of individual tumour mutations (mutanome) with great accuracy and speed. These sequencing data hold great promise for identifying unique targets and designing customized immunotherapy strategies to enhance adaptive mutation-specific immunity [340].

In the post-genome era, bioinformatic technologies and tools have been developed to identify the mutanome. Neo-epitopes can be identified by different means. Neural network algorithms, such as NetMHC, are commonly used to predict in silico the affinity of neo-epitopes derived from mutated sequences that patients bind to MHC class I molecules [341]. The predicted peptides can be synthesized and are used for dilution of patient immunity. This is called reverse identification and can generate large numbers of candidate neo-epitopes that can be further selected by bioinformatic tools based on other parameters. Currently only peptides presented in MHC-I can be predicted, as prediction of CD4 T cell epitopes by algorithms is still limited [347]. Another limitation of reverse identification is that it is not known whether neoepitopes are presented by tumour cells.

Thus, direct identification by MHC ligandome analysis of tumour cells is possible. This requires elution of the peptides presented in MHC molecules derived from the patient’s tumour tissue, reverse-phase HPLC fractionation and mass spectrometry. This approach allows the identification of CD8+ and CD4+ T cell neo-epitopes, although validation with exome and transcriptome sequencing data is still required. Although the sensitivity of neoantigen identification by this method has not yet been improved, it is likely to be a very important tool in antigen discovery in the near future [340].

To demonstrate the potential of candidate peptides for neoantigens, experimental validation of their immunogenicity using autologous T cells from the patient is necessary. Functional assays of T cells can be performed with short peptides predicted in silica for CD8+ T cells, or long peptides and mRNA for CD8+ or CD4+ T cells. Several studies have detected neoantigen-specific CD4+ T cells whose induction can control the tumour and, in murine models, the spread of antigen [344].

One strategy for identifying neo-antigens from immunogenic T cells is based on transfecting autologous APCs with RNA or DNA encoding a 12-amino acid mutated gene sequence. The APCs are incubated with autologous T cells (TILs) and the responding T cells can propagate. It is important to define the minimum length of T-cell epitopes and their MHC restriction. For this purpose, APCs expressing only one MHC molecule must be used and pulsed with predicted synthetic peptides. The problem is that large quantities of autologous T cells are required and a massive expansion of these cells.

There are currently several active clinical trials of cancer vaccines targeting neoantigens following different strategies, including poly-epitopic RNA and peptide vaccines based on high-throughput sequencing (HTS) and in silico prediction for metastatic melanoma, peptide vaccines based on HTS data with MS data in glioblastoma and poly-epitope plasmid DNA and RNA vaccines in triple-negative breast cancer.

Mutated T cells are an important component of TILs that spread in vivo and can be used for ACT in patients with melanoma. In addition, neoantigens can be used in passive immunotherapy strategies. Neoantigen-specific T cells can be isolated and expanded to re-infuse into patients. Genetically modified T cells with neoantigen-specific CRT or CAR may also be used [340].

Despite these advances, this type of personalised immunotherapy, which targets unique mutations, has limitations. Most of these mutations are unique to each specific tumour and must be identified and validated in each patient, which requires the development of massive methodologies. Later on, in silico analysis will overcome the need for massive peptide screening. Another important factor is to identify driver mutations, which are the ideal targets for cancer immunotherapy, since they are critical for tumour development, but in most tumours these mutations have not yet been identified. Therefore, the identification of neo-antigens is one of the great challenges of current immunotherapy to develop effective personalised treatments.

### 5.6. Microsatellite-Unstable Tumours

As described above, it is believed that the set of neoantigens in a tumour is highly individual. This characteristic is what differentiates them from tissue-specific antigens, tumour-associated antigens (TAAs) or other tumour-selective antigens that are considered to be shared. The exception to this is some cancers that possess a high mutational load, including microsatellite-unstable tumours, which have shared neo-antigens due to preferential mutations of several genetic regions called microsatellites.

Cancers with hereditary defects in genes involved in DNA repair have high frequencies of non-synonymous mutations, resulting in a wide variety of tumour neoantigens. In some cases, insertions and deletions also accumulate in DNA hot spots that are prone to mutations with repeating base-pair sequences, called microsatellite instability (MSI). This has made it possible to identify genes with a high mutation frequency in patients with MSI. These recurrent neoantigens can be prime targets for immunotherapy, particularly frame-shifting mutations containing multiple new epitopes that are recognised by several MHC haplotypes [348]. Patients with these types of defects are highly mutational and therefore form immune escape variants. Defects in antigenic presentation by MHC molecules and in molecules associated with MHC expression have been found in patients with MSI tumours [349]. Unstable microsatellite tumours are recognised as a subset of tumours with different prognostic and predictive characteristics.

### 5.7. Immunopeptidome as Biomarkers

The presentation of peptides in MHC molecules is a mechanism that allows the adaptive immune system to differentiate healthy cells from cancerous or infected cells.

Both MHC class I and MHC class II molecules present peptide antigens to T cells, but structurally and functionally they are different. MHC-I is constitutively expressed on all nucleated cells, including tumour cells. Despite this, some tumour cells may lose the expression of MHC-I as an immune escape mechanism. Loss of MHC-I expression is usually a trigger for NK-mediated cell death, but many tumours can still evade immune-vigilance without MHC-I expression [350]. Loss of MHC-I expression has also been reported as a mechanism of resistance to anti-PD-1 therapy [351]. Some of the tumours that lose expression of MHC-I, maintain expression of MHC-II, but their functional significance in these cases is unclear [352]. In addition, some melanoma cell lines that do not express MHC-II have high levels of MHC-I expression [353]. This suggests that MHC-I and MHC-II are independently regulated in cancer and their expression may have different implications for cancer immunotherapy [350]. In this review we will only consider the peptide load presented by MHC molecules, regardless of possible changes in expression of these molecules.

**Figure 15:**
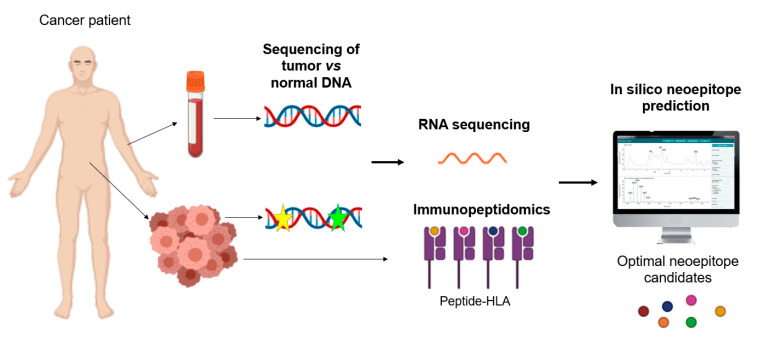
Schematic representation of tumour neoantigen screening: non-synonymous mutations determined by whole-exome or genome sequencing of tumour. These mutations can then be filtered by expression (RNA sequencing) or presentation (immunopeptidomics). In silico algorithms are used to predict and filter optimal neoepitope candidates.

The antigenic processing machinery is complex and flexible, so predictions using binding motifs or binding affinities for the deduction of MHC-restricted peptides are complicated. MHC peptidomics data based on mass spectrometry and training of prediction algorithms have led to improvements in MHC binding prediction. The precise identification and selection of new MHC peptides as targets for cancer immunotherapy must be an integrated, comprehensive and deep mapping analysis of MHC binding in both healthy and pathological tissues of all types (Figure 15). The depth of the analysis is important since antigen-specific immunotherapies are restricted to certain MHC haplotypes, reducing the fraction of detected ligands that will bind to the same MHC. Once the possible ligands have been defined, their tumour specificity must be confirmed in vivo. Neoantigens already fulfil this characteristic in themselves [354].

When these targets are selected and validated, predictive biomarkers need to be defined that allow for the identification of positive target patients with the aim of personalizing therapy and treating only those patients who can obtain clinical benefit [355]. This is performed by quantifying mRNA expression using qPCR, since it is assumed that MHC peptide presentation and mRNA expression are correlated. It has been shown that very abundant transcripts generally result in a greater number of MHC class I peptides bound [356], but this is not necessarily true for each peptide-mRNA pair [357]. Mass spectrometry is used to establish the association between peptide presentation and mRNA expression for individual peptides. In addition, this association can be translated from LC-MS/MS to RNA-seq for qPCR data, with the aim of defining predictive biomarkers that allow for precision medicine through the personalised analysis of immunopeptidome-guided mRNA expression, as shown in the study by Jens Fritsche et al. [354].

Briefly, mass spectrometry allows in-depth analysis of human immunopeptidome, expanding the number of targets available for immunotherapy. In addition, it allows the identification of predictive biomarkers based on mRNA, which can be used as complementary diagnostics to qPCR to define positive populations for the target peptide, being able to establish a personalised peptidomic. These biomarkers will improve the efficacy of precision treatment in cancer immunotherapies.

As mentioned above, MHC peptidomes have been studied to identify cancer-specific peptides, for the development of tumour immunotherapies and as a source of information on protein synthesis and degradation patterns within tumour cells. However, it is also known that the levels of soluble MHC class I molecules (sMHC-I) are increased in serum of people affected by pathologies such as cancer, autoimmunity, allergy or viral infections. In the study by Michal Bassani-Sternberg et al. [358] it was postulated that if a significant proportion of sMHC molecules in plasma are released from diseased cells (multiple myeloma, acute myeloid leukaemia and acute lymphoblastic leukaemia) and also carry their original peptide load, analysis of sMHC peptidomes may be an ideal source of biomarkers in various diseases, considering that sMHC peptidomes are similar to membrane MHC peptidomes.

It has been concluded that sMHCs carry defined sets of peptides derived primarily from tumour cells. sMHCs are released from healthy and pathological cells, so it is expected that as the patient’s tumour load increases, larger fractions of sMHC peptidomas will originate in plasma from tumour cells. Tumour cells release more sMHC into the circulation than healthy cells, possibly to evade the T-cell immune response. Diseased cells are expected to contribute differently to sMHC peptidomas, depending on the size of the tumour, its type, and its tendency to release sMHC into the circulation. An escape mechanism from immune surveillance related to elevated sMHC levels has also been described, and this characterisation would help to identify peptides involved in this mechanism [359]. Finally, analysis of sMHC peptidomas resulted in the identification of thousands of peptides, including potential biomarkers of disease that would need to be validated clinically [358].

Following this line, the Human Proteome Organization initiated the Human Immuno-Peptidome Project (HUPO-HIPP) in 2015 as a new initiative of the Biology/Disease-Human Proteome Project (B/D-HPP) with the final goal of mapping the entire repertoire of peptides presented at MHC, using mass spectrometry. In addition, this analysis is intended to be accessible to any researcher. The basis of this project is the development of methods and technologies, standardization, effective data exchange and education [360].

Currently there are already multiple databases that collect data on the immunopeptidome presented at MHC analysed by mass spectrometry. One of these is the SysteMHC Atlas (https://systemhcatlas.org), which contains raw immunopeptidomics MS data. They are processed through bioinformatic tools to identify, annotate and validate the peptides in MHC. This generates libraries with the lists of MHC peptide ligands and allele and sample specific peptide spectra. Each project is labelled as HIPP, being a sub-project of B/D-HPP [361].

The aim of this type of database is to allow basic scientists and clinicians to access a large catalogue of MHC-associated peptides to obtain new ideas about the composition of the immunopeptidome; to allow computational scientists to use this data to develop new algorithms for immunopeptidomic analysis; and to allow access to these libraries to facilitate analysis by next-generation MS [361].

Peptide binding affinity prediction tools allow for the detection of peptides in silica for the purpose of identifying T cell epitopes that match MHC-II molecules from a particular host. One of these tools are NetMHCII and NetMHCIIpan [362,363], which are based on ensembles of artificial neural networks that are trained on quantitative peptide binding affinity data from Immune Epitope Database (IEDB) [364]. These types of tools can improve MHC-II binding predictions and reduce experimental costs in epitope-based vaccine design. Understanding peptide–MHC interactions is key to the cellular immune response.

### 5.8. TAAs as Biomarkers

Autoantibodies are useful biomarkers in clinical diagnosis and are biological agents used to isolate and study the function of intracellular molecules that are self-antigens targets [365]. In other words, they make it possible to characterise their related antigens and clarify the pathogenic mechanisms. These autoantibodies are found in autoimmune diseases but also in cancer [366]. Antibodies to tumour-associated antigens (TAA) in cancer are similar to autoantibodies detected in systemic autoimmune diseases, as these anti-TAAs also have the potential to be diagnostic markers in cancer. The identification of these TAAs in cancer patients allows the study of the mechanisms by which molecular and other alterations of intracellular proteins drive autoimmune responses. Many of the TAAs identified by the autoantibodies of cancer patients have important cellular biosynthetic functions that may be related to carcinogenesis [367]. In addition, there are autoantibody profiles that are unique to each type of cancer and other antibodies are shared. These profiles may serve as diagnostic markers in cancer [365,368].

An example is autoantibodies to p53, which report early carcinogenesis [369]. Anti-p53 has been detected in chronic obstructive pulmonary disease [370], which is prone to the development of lung cancer, as well as in workers exposed to vinyl chloride, who have the same predisposition [371].

Autoantibodies to TAA have also been found in hepatocellular carcinoma [372]. Chronic hepatitis and liver cirrhosis are precursors of hepatocellular carcinoma [367]. After collecting samples from patients with these diseases, the target antigens for antibodies associated with malignancy have been detected. These autoantibodies are usually antinuclear antibodies, and in this case the antigens detected were topoisomer II DNA-α and -β [373]. In addition, in cases where antigens have been identified, these were molecules involved in cell proliferation and gene regulation. Therefore, the patients’ immune system appears to be reacting to these factors involved in carcinogenesis [367].

One of the characteristics of TAAs is that they have functions involved in proliferation, transformation and other processes associated with malignancy. Although these molecules are present in most cell types, any alteration of their normal state will only be detectable in malignant cells.

In order to understand how these TAAs acquire immunogenicity, studies have been performed with different TAAs identified, such as p53, p62 or cyclin B1 [367,374]. p62 auto-antibodies to these molecules were detected in patients with hepatocellular carcinoma and their expression was also studied in patients with liver cirrhosis, normal liver biopsies and fetal liver samples. What is observed is that p62 is developmentally regulated and expressed in the fetal liver, but not in the adult, except in malignant liver cells, where it is expressed aberrantly, suggesting that this TAA is an oncofetal antigen [367]. Cyclin B1 has been identified as TAA in patients with hepatocellular carcinoma because it is present in both B-cell and T-cell immune responses and is known to play an important role in the progression of the cell cycle from G2 to M [375].

The importance of TAAs and their autoantibodies in cancer has been seen in both diagnosis and surveillance and in therapy. Many approaches therefore focus on identifying a large number of TAAs, including proteomic approaches such as protein microarrays [367].

To track and understand human autoantigens and to conduct basic and translational research on their functions, databases of human autoantigens have been created. These include AAgAtlas 1.0 (http://biokb.ncpsb.org/aagatlas). This database provides an interface to explore and download human autoantigens and their associated diseases. Human autoantigenic proteins are involved in major diseases, such as the immune system, hypersensitivity reaction or cancer, so these databases are an effective tool to investigate the functions of these proteins and to develop future immunotherapies [376].

Autoantibodies have been integrated as biomarkers with different proteins and are the first protein-based blood test that allows the early detection of cancer after the development of the Videssa^®^ Breast Kit, as discussed above, for breast cancer [306].

Ultimately, cancer immunotherapy is based on the use of peptide antigens derived from amino acid sequences of tumour antigens, focusing on modulating the response of T cells [377]. One of the problems is selecting candidate peptides, since they must be strongly immunogenic to induce the desired response by T cells. To do this, it is important to identify regions of AAD that are recognised by the patient’s immune system in order to indicate realistic targets in vivo and to design an immunotherapy that targets these auto-epitopes [367]. These T cell auto-epitopes can be identified from MHC class I molecules [378].

### 5.9. Techniques for Biomarkers Discovery, Verification and Validation

As we have seen, mass spectrometry is a powerful technology in biological research and allows the characterisation of the plasma proteome in great depth. This has been performed using “triangular strategies”, which aim to discover unique biomarker candidates in small cohorts, followed by classical immunoassays in larger validation cohorts. Currently, a “rectangular strategy” is proposed, in which the proteome patterns of large cohorts are correlated with their phenotypes in health and disease. The methodologies developed for biomarker detection are described below [351].

Mass spectrometry (MS) measures the mass spectra and fragmentation of peptides derived from protein digestion very precisely. These sequences are unique, so proteomics is very specific, unlike enzymatic colorimetric tests or immunoassays [379]. In addition, MS allows the analysis of the entire proteome and the quantification of post-translational modifications (PTM). The discovery of PMTs is important as they can form the basis of diagnostic tests.

So far, none of the laboratory tests routinely performed are based on proteins that have been identified by MS and only small molecules (drugs, metabolites) have been used in MS technology [380].

MS has improved its performance in dynamic range and sensitivity, making it optimal for the study of biomarkers. Currently, plasma proteins are the type of molecules most frequently analysed in the clinic, using enzymatic reactions or antibody immunoassays. These methods are the ones selected in the clinic because the time required for the analysis is a few minutes. The main advantage of MS-based proteomics is that it is not necessary to assume the nature or number of potential biomarkers. This strategy allows all possible biomarker studies to be combined for each disease and their relationship to each other [351].

A “triangular strategy” has been proposed to identify biomarkers. This strategy is composed of different steps, in which the number of individuals is increased and the number of proteins is gradually decreased [380]. The first step is to identify peptides following the workflow for hypothesis-free discovery proteomics: enzymatic digestion of proteins by HPLC and peptide analysis by MS/MS, followed by the use of proteomics software platforms to identify and quantify these peptides. The second phase of the strategy triangulates the verification of candidates. In this case, a low number of candidate proteins are tested, selecting a set of peptides, in a larger cohort. Multiple reaction monitoring methods (MRM) are used and only those peptides chosen are fragmented, so that they can be quantified with high sensitivity and specifically. In the third phase, validation with sandwich immunoassays is performed, as they are very specific and have high sensitivity. In this case, only a few candidate biomarkers are validated in a cohort that may consist of thousands of patients [351].

Thanks to the improvements in the LC-MS/MS system and the robustness of bioinformatic analysis, and with the aim of developing a fast and automated workflow that quantifies in depth the plasma proteome in a large number of samples, a “rectangular strategy” has been proposed. In this case, the aim is to measure as many proteins as possible for all possible individuals and conditions. For this purpose, the initial cohort would be much larger and would allow for the identification of significant differences in the proteins. Cohort discovery and validation can be measured by shotgun proteomics, allowing both cohorts to be analysed at the same time. This strategy has the advantage of being able to discover and validate protein patterns characteristic of specific health or disease states and unique biomarker candidates [351].

The goal in proteomics is to achieve sufficient depth in a short time, without exhaustion and with a robust workflow, to allow for the identification of unique biomarkers that can be used in the clinic.

## 6. Conclusions and Perspectives

The development of anti-cancer drugs has focused on strategies that kill cancer cells directly, such as surgery, radiotherapy, chemotherapy, targeted therapy and immunotherapy. Immunotherapy is based on the recognition of tumour cells as foreign by the immune system. For good results, immunotherapy has to activate and expand tumour-specific T cells. Various approaches have been used for this purpose: direct activation of anti-tumour immunity by means of cancer vaccines (tumour antigens), recombinant cytokines or the infusion of tumour-specific cells. However, these methods did not guarantee that the tumour-specific T cells could nest or perform their function within the tumour. This is due to the existence of tumour-induced immunosuppressive mechanisms in the tumour microenvironment, which prevent the breakdown of immune tolerance to cancer. With the approval of the checkpoint inhibitors, it was demonstrated that an anti-tumour immune response can be initiated by targeting the immune system to break the tolerance to cancer. In contrast, a significant clinical response was only obtained in a few patients with solid tumours such as melanoma, non-small cell lung cancer, kidney or bladder cancer. This is because the response to this treatment depends on the presence of pre-existing tumour-specific CD8 T cells, which correlates with the presence of neoantigens derived from tumour mutations. Because of this relationship, different approaches have been tested that allow for the expansion of anti-tumour T cells along with checkpoint inhibition. These combination therapies have been successful in clinical trials, but the current focus is on targeted therapies to target neoantigens derived from tumour mutations, as the number of mutations in each tumour correlates directly with the efficacy of checkpoint inhibitors. Therefore, it is necessary to establish combined therapies in cancer, among which small molecule inhibitors also stand out. These combination approaches are key to understanding the relationship between the established tumour and the immune system [381]. Combination therapies are useful in inhibiting tumour growth and changing or restoring the TME. Among the most explored are combinations of checkpoint inhibitor (anti-PD1) and targeted (antibody or small molecule) therapies. Rapid lysis of tumour cells with targeted therapies can generate an environment of acute inflammation that enhances tumour immunity, making these therapies additive [73].

On the other hand, programmed physiological cell death, usually in the form of apoptosis, has always been considered a non-immunogenic or even tolerable process. In contrast, the concept of “programmed cell death” (PCD) has assigned immunogenic capabilities to apoptosis. This type of apoptosis is characterised by the ability of dying cells to trigger adaptive immune responses against the altered autoantigens/neo-epitopes derived from cancer, in the case of tumour cells. In addition to antigenicity, adjuvancy, conferred by DAMPs, is necessary.

The pathways that induce ICD can be used to design new therapeutic tools in immunotherapy, to reduce the tumour burden and improve the immunogenic capacity of dying tumour cells, provoking adaptive immune responses in the long term. Various immune-based therapies can benefit from ICD, such as antibody-based therapies, adoptive cell therapy (TIL, NK cell or CAR-T), checkpoint inhibitors, tumour vaccines and combination immunotherapy strategies [256]. One of the most promising strategies is to exploit the ICD concept to obtain highly immunogenic antigen sources for the development of “next generation” DC-based vaccines [382]. ICD inducers can be used to generate immunogenicity in dying tumour cells and to load DC, enhancing their ability to stimulate effector cells and improve T-cell responses to cancer in vivo [99]. This may improve general immunity or create an immune-friendly tumour microenvironment [256]. A number of chemotherapeutic agents are ICD inducers, meaning that many therapeutic strategies have known immunomodulatory or immune-stimulatory effects that should be further investigated to determine if they are associated with the release of DAMPs. The characterisation of new DAMPs may open up new therapeutic targets for targeted chemotherapy [383]. Understanding the molecular pathways involved in these processes would allow for the identification of a new set of potential prognostic biomarkers, but more research is needed to understand the true impact of ICD therapy and exposure to DAMPs [256].

The immune system of the cancer patient detects abnormalities in structure, function, intracellular location, and other cellular alterations during tumorigenesis, which may manifest themselves in humoral or cellular immune responses, which may be the earliest sign of carcinogenesis. The current aim is to use cancer autoantibodies as diagnostic biomarkers, but there is also the possibility that they may be used as monitors of the therapeutic response. If an anti-TAA antibody is detected in the patient, changes in the levels of these antibodies may reflect the status of the tumour, its changes or its tumour load in relation to therapy [367].

Cancer immunotherapy relies heavily on the use of peptide antigens derived from amino acid sequences of tumour antigens and modulates the response of T cells. The problem here is that the peptides selected must be strongly immunogenic and induce a T cell response. Therefore, it would be important to identify the regions of the TAAs that can be recognised by the patient’s immune system, which would confirm that they are real targets in vivo and allow for the design of immunotherapies directed to these auto-epitopes. Such auto-epitopes must be able to be identified and isolated from MHC class I molecules [367].

In this sense, T lymphocytes αβ detect alterations in the host’s cellular components, which may be induced by infectious pathogens, chemical or physical damage or oncogenic transformation. The T-lymphocytes generated in the thymus each have a clonally restricted T cell receptor (TCR) [384]. Human tumours contain a high number of somatic mutations, and if peptides containing these mutations occur on MHC class I molecules they may be immunogenic and recognised by the adaptive immune system, which recognizes them as “non-self” neoantigens. Mutant peptides can serve as T cell epitopes [385]. During immune surveillance, each T cell receptor recognizes a different foreign peptide attached to MHC molecules. MS technology allows for the identification of epitopes relevant to different tumours. Molecular CCR cloning methods allow for the molecular quantification of TCR–pMHC interactions [384,386]. This is a major challenge, since MHC-bound peptidoma consists of thousands of different peptides with relevant non-self-antigens often embedded in low numbers, among them the self-peptides, which occur in a greater order of magnitude [384].

In immunogenic tumours, the sequencing of the complete exome and transcriptome of individual tumours, together with mass spectrometry, allows for the identification of mutant peptides to develop vaccines on an individual basis for each patient [385]. In non-immunogenic tumours, the induction of the expression of multiple neoepitopes can direct a polyclonal CTL attack against a cancer. One goal of therapeutic antitumour vaccines is the targeting of CTLs on MHC-bound peptides restricted to cancer cells, increasing the CTLs of high avidity at the site of the tumour [386].

In contrast, few mutant epitopes have been described, as it requires the exploration of the patient’s tumour infiltrating lymphocytes based on their ability to recognize antigen libraries created after sequencing of the tumour exome. This requires the use of mass spectrometry combined with transcriptomical or exome sequence analysis to identify neo-epitopes [385].

In short, a large part of the antigens that drive the effectual responses of antitumour CD8 T cells remains unknown. These antigens can be classified into tumour-associated autoantigens and antigens derived from tumour-specific mutant proteins. The presentation of autoantigens in the thymus may result in the elimination of highly avid T cells, thus mutant neo-antigens will be more immunogenic. In contrast, these neoantigens evade identification by mass spectrometry because this method relies on sequence clarification with proteomic databases that do not contain patient-specific mutations. By using transcriptomics and exome sequence analysis to identify mutations, together with the use of MHC class I binding prediction algorithms, too many candidate mutant peptides are detected to be evaluated. Mass spectrometry would allow for the selection of peptides with sufficient expression and presentation by MHC class I, which would be the most immunogenic. By combining both tools, it is possible to identify mutated peptides associated with tumours that present in MHC class I [385].

The immunogenicity of neoepitopes is correlated with the affinity for peptide binding by MHC class I, but other factors such as the interaction of the mutated amino acid with the TCR also play a role, as this is essential for the recognition of the mutated peptide as a stranger [387].

The analysis of MHC peptidoma allows for the identification of peptides derived from the proteolysis of proteins that are generally short-lived in tumour tissues and therefore cannot be identified by conventional proteomic methodologies. One of the current challenges is to find differences between MHC peptidomas from healthy patients and cancer patients, since MHC peptidomas have large amounts of different peptides. Most of these peptides will be from the cellular proteome but also small amounts of cancer-related peptides will be present, which will be different even among different patients presenting the disease. Ultimately, such analysis would allow for the identification of thousands of peptides, including some potential biomarkers of disease. Furthermore, these cancer-related MHC peptides could be used to design patient-specific immunotherapeutics. In other words, the final goal would be to use the MHC peptidoma data to personalize treatments. Mass spectrometry analysis would be a good tool for this purpose, as it is becoming less expensive and faster and can be used in clinical diagnosis on a routine basis [358].

In conclusion, the identification of epitopes that induce the immune response in cancer is necessary to understand and manipulate the immune responses of CD8 T cells for clinical benefit. Tumour-specific mutations are important in shaping the antitumour response, but their identification remains a challenge. The identification of neo-epitopes by combining whole exome sequencing, transcriptome and mass spectrometry analysis strategies, together with a structural prediction algorithm to predict peptide immunogenicity in MHC class I would facilitate the monitoring of tumour-specific T cells, which would be useful in the prognosis of cancer patients, as well as the development of new vaccines [385].

Cancer immunotherapy is undergoing a major transition from traditional approaches that activate systemic immune responses based on understanding the processes of immune activation to more effective and less toxic treatments that target immune normalization in the tumour microenvironment based on tumour-induced immune escape mechanisms [73].

NPs play an important role in these improvements in immunotherapeutic treatments. Nanomaterials applied to nanomedicine would make it possible to increase the effectiveness and reduce the toxicity of practically all the immunotherapeutics described. There are very varied nanoparticle designs that can serve as immunotherapy delivery platforms, allowing for specific and targeted delivery. In addition, NPs can also be designed to enhance the immune response of the host. Due to their great potential, research into these nanomaterials in combination with drugs is necessary to ensure their biosafety and determine their specific functions and applications based on their biocompatibility. Improving these approaches would make it possible to overcome some of the drawbacks of immunotherapy and initiate a new path in cancer treatment.

## Figures and Tables

**Table 1:** Molecules that act as Damage-Associated Molecular Pattern (DAMPs), associated pattern recognition receptors (PRRs) and described biological functions.

Danger Signal	PRR	Function
CALR HSP70 HSP90	LRP1	Promotes the uptake of dead cell-asociated antigens.
Extracellular ATP	P2RX7/P2RY2	Favours the recruitment of APCs and their activation.
HMGB1 dsRNA Cellular RNA LPS Flagellin ssRNA CpG DNA Viral RNA dsDNA	TLR2/TLR4 TLR3 TLR3 TLR4 TLR5 TLR7 TLR9 RLRs CDSs	Activate the synthesis of pro-inflammatory factors (type I IFNs).
Type I IFNs	IFNAR	Promotes CXCL10 secretion by cancer cells and has immune-stimulatory effects.
ANXA1	FPR1	Guides the final approach of APCs to dying cells.
CXCL10	CXCR3	Favours T cell recruitment.

Caption: ANXA1, annexin A1; APC, antigen-presenting cell; CALR, calreticulin; CDS, cytosolic DNA sensor; CXCL10, CXC-chemokine ligand 10; CXCR3, CXC-chemokine receptor 3; ds, double-stranded; FPR1, formyl peptide receptor 1; HMGB1, high-mobility group box 1; HSP70, heat shock protein 70 kDa; HSP90, heat shock protein 90 kDa; IFN, interferon; IFNAR, interferon α/β-receptor; LRP1, LDL receptor related protein 1; P2RX7, purinergic receptor P2X7; P2RY2, purinergic receptor P2Y2; RLR, RIG-I-like receptor; ss, single-stranded; TLR, Toll-like recept.
